# Incidence and risk factors of acute kidney injury following Stanford type A aortic dissection surgery: a systematic review and meta-analysis

**DOI:** 10.3389/fcvm.2026.1854958

**Published:** 2026-06-15

**Authors:** Yu Yang, Xiaojia Shan, Xiaowei Tang, Yiwen Ding, Xuemei An

**Affiliations:** 1 College of Nursing, Chengdu University of Traditional Chinese Medicine, Chengdu, China; 2 Department of Nursing, Hospital of Chengdu University of Traditional Chinese Medicine, Chengdu, China; 3 Administrative Office, Deyang Hospital, Affiliated Hospital of Chengdu University of Traditional Chinese Medicine, Deyang, China

**Keywords:** acute kidney injury, incidence, meta-analysis, risk factors, Stanford type A aortic dissection, systematic review

## Abstract

**Background:**

This study aims to synthesize the existing evidence to identify the incidence of AKI following surgery for TAAD and its primary risk factors.

**Methods:**

Systematically searched PubMed, Embase, Web of Science, the Cochrane Library, CNKI, Wanfang, VIP, and the China Biomedical Literature Database to identify studies published from the inception of each database through January 6, 2026, regarding the incidence and risk factors of postoperative AKI following TAAD. Two researchers independently screened the literature and extracted data, and the Newcastle-Ottawa Scale (NOS) was used to assess study quality. Meta-analysis was performed using Stata 15.0 software.

**Results:**

A total of 44 studies were included, with a total sample size of 11,983 patients, of whom 6,115 developed AKI. The meta-analysis showed that the overall incidence of postoperative AKI following TAAD was 50.72%. Subgroup analysis showed that the incidence rate was higher when diagnosed using the KDIGO criteria compared to the AKIN and RIFLE criteria; the incidence rate in Chinese studies was higher than that in non-Chinese studies. Regarding risk factors, age (per 1-year: OR = 1.03; per 10-year: OR = 1.40), male (OR = 1.72), BMI (OR = 1.13), history of hypertension (OR = 1.59), preoperative serum creatinine (OR = 1.02), preoperative leukocyte count (OR = 1.04), preoperative lactate (OR = 1.28), renal artery involvement (OR = 3.47), cardiopulmonary bypass time (per 1-minute increase: OR = 1.01), operative time (OR = 1.33), deep hypothermic circulatory arrest time (OR = 1.07), red blood cell transfusion volume (OR = 1.18), and duration of mechanical ventilation (OR = 1.01) may be significantly associated with postoperative AKI following TAAD.

**Conclusion:**

The incidence of AKI following TAAD is high, with major risk factors including advanced age, male gender, high BMI, hypertension, elevated preoperative serum creatinine and white blood cell count, renal artery involvement, prolonged cardiopulmonary bypass and deep hypothermic circulatory arrest, increased perioperative blood transfusion, and prolonged mechanical ventilation. Clinically, a comprehensive prevention and control system covering preoperative assessment, intraoperative protection, and postoperative monitoring should be established to reduce the incidence of AKI and improve patient outcomes.

**Systematic Review Registration:**

https://www.crd.york.ac.uk/prospero/, PROSPERO CRD420261276938.

## 1 Introduction

Aortic dissection (AD) is a rapidly progressing cardiovascular emergency with a high mortality rate (1). The main pathological feature is acute injury to the aortic intima, where high-speed blood flow penetrates the tear in the intima and enters the aortic media, propagating longitudinally along the aorta, resulting in pathological separation between the true and false lumens (2). Based on whether the ascending aorta is involved, it can be classified into Stanford A and B types. Stanford A type aortic dissection (TAAD) accounts for approximately 58%–62% of all AD cases (3). Despite recent advancements in imaging diagnosis and perioperative management, the overall prognosis of TAAD remains poor. Previous studies have shown that the mortality risk of untreated TAAD patients increases rapidly over time. Without surgical repair, the estimated mortality rate is about 1%–2% per hour, with a mortality rate of up to 50% within the first 24 h (4). Therefore, emergency surgical repair is still considered the most effective and only gold standard treatment method at present (5). TAAD surgery typically involves deep hypothermic circulatory arrest, cardiopulmonary bypass (CPB), and complex aortic reconstruction, which significantly affects the function of vital organs throughout the body, and the incidence of postoperative complications remains high (5–7).

Acute kidney injury (AKI) is a syndrome characterized by a rapid decline in renal function within a short period of time, accompanied by elevated serum creatinine or decreased urine output (8). AKI is one of the early postoperative complications of TAAD and a major cause of poor prognosis after cardiac surgery, associated with subsequent chronic kidney disease and adverse cardiovascular events (9). Existing studies have shown that the incidence of AKI after TAAD surgery is high and varies significantly, ranging from approximately 20% to 86% in different reports (10, 11), with about 5% to 40% of patients requiring renal replacement therapy (12, 13). Research has found that postoperative AKI is also associated with prolonged hospital stays, increased likelihood of readmission, high in-hospital mortality, decreased long-term survival, and increased medical burden (14–16).

Given the significant impact of AKI on the prognosis of patients with TAAD, early identification of its risk factors and implementation of targeted interventions are of great clinical significance. In recent years, scholars worldwide have conducted numerous studies on the risk factors of AKI after TAAD surgery. Risk factors include patient baseline characteristics such as age and baseline renal function, intraoperative factors such as CPB time and operation time, as well as perioperative management (17, 18). However, due to significant differences in sample size, AKI diagnostic criteria, and population characteristics across different studies, current conclusions regarding the incidence of AKI after TAAD surgery and its key risk factors remain inconsistent, and even some factors show opposite results in different studies (19). This emphasizes the necessity of conducting systematic reviews and meta-analyses to integrate existing evidence to clarify the true incidence of AKI after TAAD surgery and its main influencing factors.

This study intends to comprehensively analyze recent research on the incidence and risk factors of AKI after acute Stanford A type aortic dissection surgery through systematic review and meta-analysis. It aims to facilitate more accurate identification of high-risk patients during the perioperative period, optimize surgical strategies and perioperative management, thereby reducing the incidence of AKI and improving overall patient outcomes.

## 2 Methods and materials

This systematic evaluation and meta-analysis will strictly follow the PRISMA (Preferred Reporting Items for Systematic Reviews and Meta-Analyses) guidelines (20). And it is registered in Prospero with registration number CRD420261276938.

### 2.1 Literature search

Two researchers independently searched PubMed, Embase, the Cochrane Library, Web of Science, the China Knowledge Resource Integrated Database (CNKI), the Wangfang Database, the Weipu Database (VIP), and the Chinese Biomedical Database (CBM) to identify relevant studies published up to January 6, 2026. The search strategy combined Medical Subject Headings (MeSH) and free-text terms, including “Aortic Dissection”, “Dissecting Aneurysms”, “Dissecting Aneurysm Aorta”, “Acute Kidney Injury”, “Acute Renal Injury”, and others. The detailed search strategy is presented in Supplementary Table S1.

### 2.2 Inclusion and exclusion criteria

#### 2.2.1 Inclusion criteria

1. Study Type: Prospective or retrospective observational studies (cohort studies and case control)
2. Study Population: Adult patients (age > 18) with clinically confirmed as Stanford A Aortic Dissection undergoing surgery.
3. Outcome: Studies are required to explicitly report the incidence or risk factors of postoperative AKI in patients with TAAD. Exposure factors included in the analysis are those reported in three or more studies, with largely consistent definitions across studies to ensure comparability. AKI is required to be diagnosed according to standardized criteria, including AKIN (Acute Kidney Injury Network), RIFLE (Risk, Injury, Failure, Loss of kidney function, End-stage renal disease), or KDIGO (Kidney Disease: Improving Global Outcomes). Specifically, AKI is defined as an increase in serum creatinine to ≥1.5 times the baseline within 7 days postoperatively, or an absolute increase of ≥26.5 μmol/L within 48 h.

#### 2.2.2 Exclusion criteria

1. Studies analyzing only the incidence or risk factors of postoperative acute renal failure, continuous renal replacement therapy (CRRT), or Stage 2 or 3 acute kidney injury occurring postoperatively.
2. Case reports, conference abstracts, reviews, meta-analyses, animal studies, letters, or reports without original data.
3. Articles whose full text could not be obtained.

### 2.3 Study selection and data extraction

Data extraction and cross-verification were independently performed by two researchers using Microsoft Excel. For literature with incomplete data, the authors were contacted via email to provide the missing information. Extracted data included: the first author, publication date, region, sample size, number of cases of postoperative acute kidney injury (AKI), diagnostic criteria for AKI, and risk or predictive factors for postoperative AKI.

### 2.4 Quality assessment

Two researchers independently assessed the quality of the literature using the Newcastle-Ottawa Scale (NOS) (21); any discrepancies in opinion were resolved through discussion with a third researcher. The NOS assessment covers three domains: selection of study participants (4 items, 4 points), comparability between groups (2 items, 2 points), and measurement of outcomes or exposure factors (3 items, 3 points), comprising a total of 9 items with a maximum score of 9 points. Studies with a score of ≤4 were classified as low-quality literature, those scoring 5–6 as moderate-quality, and those scoring ≥7 as high-quality.

### 2.5 Statistical analysis

Statistical analyses were performed using Stata 15.0 software. The incidence of postoperative AKI following Stanford Type A aortic dissection surgery was estimated using incidence rates (%) and their corresponding 95% confidence intervals (CIs). For risk factors suitable for meta-analysis, odds ratios (ORs) and their 95% CIs were used to quantify their association with postoperative AKI. To pool these ORs, a random-effects model was employed to synthesize the data, thereby accounting for heterogeneity among studies as well as variability in effect sizes across studies. The OR and 95% CI for each individual study were calculated and pooled to derive an overall effect size. The I² statistic was used to assess heterogeneity among the pooled studies. An *I*² value greater than 50% was considered indicative of substantial heterogeneity, necessitating further investigation into the sources of such heterogeneity. In cases of high heterogeneity, sensitivity analyses were conducted to identify potential factors that might influence the pooled effect size. To detect publication bias, funnel plots were generated and visually inspected for asymmetry. If the funnel plot exhibited signs of asymmetry, Egger's test was performed to assess the statistical significance of the bias. A *p*-value < 0.05 indicated the presence of publication bias, whereas a *p*-value > 0.05 suggested an absence of significant bias. If deemed necessary, the “trim-and-fill” method was applied to adjust for potential publication bias and to validate the robustness of the results.

## 3 Results

### 3.1 Literature retrieval results

**Figure 1:**
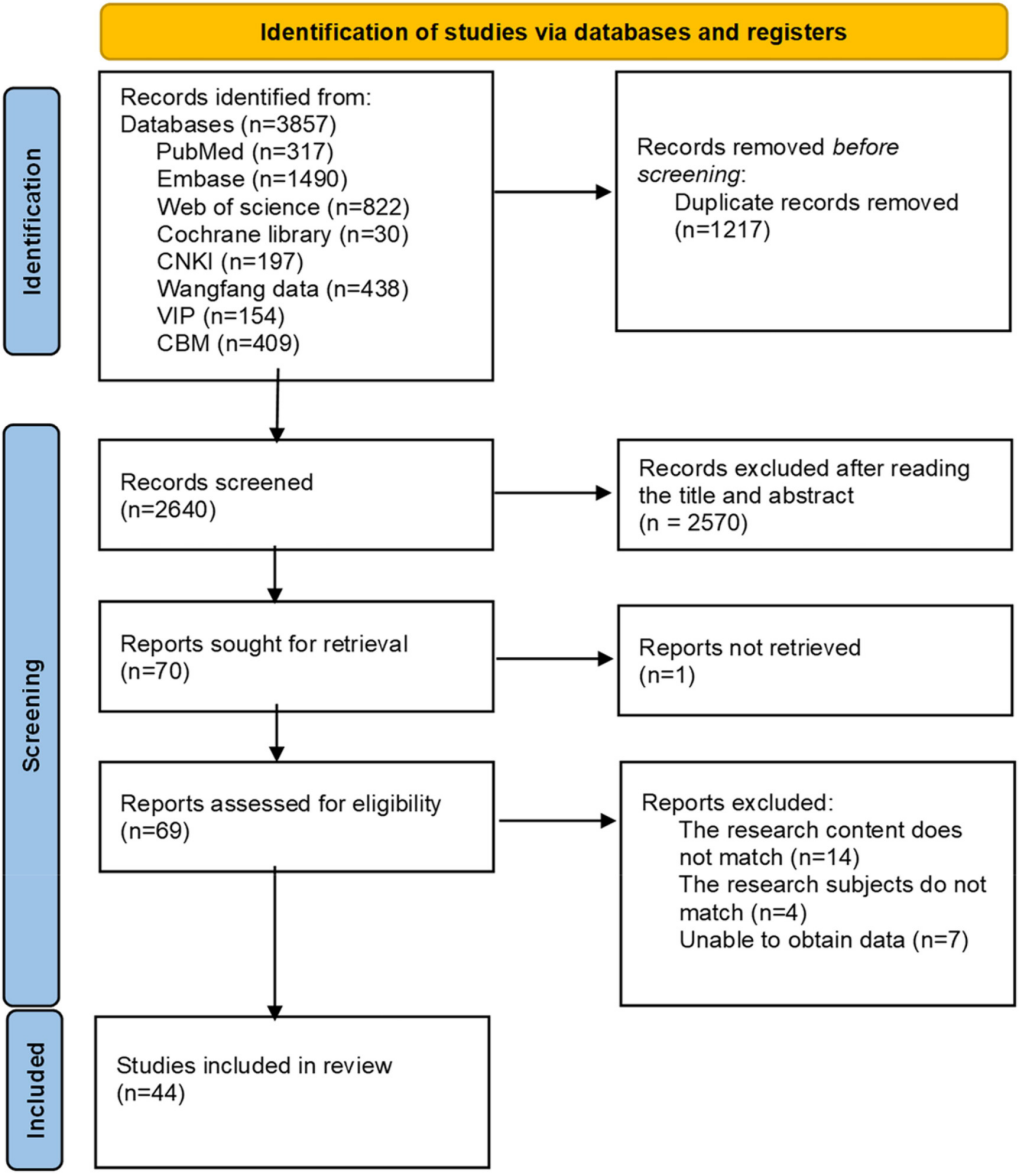
*Consider, if feasible to do so, reporting the number of records identified from each database or register searched (rather than the total number across all databases/registers. **If automation tools were used, indicate how many records were excluded by a human and how many were excluded by automation tools.

As shown in Figure 1, a total of 3,857 articles were retrieved from PubMed (*n* = 317), Embase (*n* = 1,490), Cochrane Library (*n* = 30), Web of Science (*n* = 822), CNKI (*n* = 197), Wangfang data (*n* = 438), VIP (*n* = 154) and CBM (*n* = 409). After removing 1,217 duplicate records, 2,571 articles were excluded based on title and abstract screening, 1 article was excluded due to the unavailability of full text, and 25 articles were excluded after full-text review. Ultimately, 44 articles were included.

### 3.2 Basic characteristics table of included study

All 44 included studies were cohort studies. It involved 11,983 patients, among whom 6,115 developed AKI. Detailed baseline characteristics are presented in Table 1.

**Table 1:** The basic characteristics of the included literature (*n* = 44).

Study	Year	Region	Study design	Sample size	Number of AKI	Gender (M/F)	Definition of AKI	Regression model
Song et al. (22)	2012	Zhengzhou, China	cohort study	176	78	115/61	AKIN	Logistic regression
Ruan et al. (23)	2014	Beijing, China	cohort study	169	95	129/40	KDIGO	Logistic regression
Ko et al. (24)	2015	Tokyo, Japan	cohort study	375	165	196/179	KDIGO	Logistic regression
Qiu et al. (25)	2015	Fuzhou, China	cohort study	155	56	116/39	AKIN	Logistic regression
Gong et al. (26)	2019	Beijing, China	cohort study	115	61	86/29	KDIGO	Logistic regression
Xue et al. (27)	2019	Nanjing, China	cohort study	161	73	125/36	KDIGO	Logistic regression
Fang et al. (28)	2019	Beijing, China	cohort study	627	473	476/151	KDIGO	Logistic regression
Li et al. (29)	2020	Chengdu, China	cohort study	335	241	270/65	KDIGO	Logistic regression
Liu et al. (30)	2020	Beijing, China	cohort study	88	40	57/31	KDIGO	Logistic regression
Liu et al. (31)	2020	Wuhan, China	cohort study	130	82	101/29	KDIGO	Logistic regression
Wang et al. (19)	2020	Nanjing, China	cohort study	712	359	522/450	KDIGO	Logistic regression
Zong et al. (32)	2020	Nanjing, China	cohort study	121	51	98/23	KDIGO	Logistic regression
Renati et al. (33)	2021	Urumqi, China	cohort study	175	96	142/33	KDIGO	Logistic regression
Wang et al. (34)	2021	Beijing, China	cohort study	88	37	57/31	RIFLE	Logistic regression
Zhou et al. (35)	2021	Urumqi, China	cohort study	131	64	109/22	KDIGO	Logistic regression
Helgason et al. (17)	2021	Denmark, Finland, Iceland, Switzerland	cohort study	941	382	631/310	RIFLE	Logistic regression
Tong et al. (36)	2021	Beijing, China	cohort study	660	297	535/125	KDIGO	Logistic regression
Zhang et al. (11)	2021	Shijiazhuang, China	cohort study	60	52	43/17	KDIGO	Logistic regression
Chen et al. (37)	2022	Nanjing, China	cohort study	100	38	62/38	KDIGO	Logistic regression
Li et al. (38)	2022	Nanjing, China	cohort study	211	123	161/50	KDIGO	Logistic regression
Yang et al. (39)	2022	Xi'an, China	cohort study	143	73	121/22	KDIGO	Logistic regression
Chen et al. (40)	2022	Nanjing, China	cohort study	159	47	118/41	KDIGO	Logistic regression
Li et al. (41)	2022	Beijing, China	cohort study	421	228	327/94	KDIGO	Logistic regression
Yang et al. (42)	2022	Xi'an, China	cohort study	398	268	318/80	KDIGO	Logistic regression
Li et al. (43)	2023	Urumqi, China	cohort study	176	147	152/24	KDIGO	Logistic regression
Xiao et al. (44)	2023	Chongqing, China	cohort study	111	69	88/23	KDIGO	Logistic regression
Xu et al. (45)	2023	Beijing, China	cohort study	817	305	598/219	KDIGO	Logistic regression
Chen et al. (46)	2023	Beijing, China	cohort study	382	153	277/105	KDIGO	Logistic regression
Guan et al. (47)	2023	Beijing, China	cohort study	160	84	114/46	KDIGO	Logistic regression
Xu et al. (18)	2023	Fuzhou, China	cohort study	254	74	176/78	KDIGO	Logistic regression
Xu et al. (48)	2023	Beijing, China	cohort study	624	235	460/164	KDIGO	Logistic regression
Lei et al. (49)	2024	Zhengzhou, China	cohort study	102	47	71/31	KDIGO	Logistic regression
Li et al. (50)	2024	Beijing, China	cohort study	86	44	62/24	KDIGO	Logistic regression
Abdulwahab et al. (10)	2024	Aachen, Germany	cohort study	143	29	96/47	KDIGO	Logistic regression
Cai et al. (51)	2024	Fuzhou, China	cohort study	159	54	123/36	KDIGO	Logistic regression
Jiang et al. (52)	2024	Guangzhou, China	cohort study	396	315	326/70	KDIGO	Logistic regression
Li et al. (53)	2025	Xuzhou, China	cohort study	183	84	83/100	KDIGO	Logistic regression
Liu et al. (54)	2025	Xi'an, China	cohort study	133	55	69/64	KDIGO	Logistic regression
Xu et al. (55)	2025	Chongqing, China	cohort study	138	95	104/34	KDIGO	Logistic regression
Zhang et al. (56)	2025	Bengbu, China	cohort study	80	19	53/27	KDIGO	Logistic regression
Chen et al. (57)	2025	Nanjing, China	cohort study	543	339	412/131	KDIGO	Logistic regression
Li et al. (58)	2025	Changsha, China	cohort study	447	262	351/96	KDIGO	Logistic regression
Wang et al. (59)	2025	Shanghai, China	cohort study	224	155	166/58	KDIGO	Logistic regression
Zhao et al. (60)	2025	Chengdu, China	cohort study	174	71	154/20	KDIGO	Logistic regression

### 3.3 Risk of bias results

As shown in Table 2, 29 studies scored 9 points, 12 studies scored 8 points, and 3 studies scored 7 points. All articles included in this study were of high quality.

**Table 2:** NOS scores results.

Cohort study									
References	Representativeness of the exposed group	Selection of non-exposed groups	Determination of exposure factors	Identification of outcome indicators not yet to be observed at study entry	Comparability of exposed and unexposed groups considered in design and statistical analysis	Design and statistical analysis	Adequacy of the study's evaluation of the outcome	Adequacy of follow-up in exposed and unexposed groups	Total scores
Song et al. (22)2)	*	*	*	/	*	*	*	*	7
Ruan et al. (23)	*	*	*	*	**	*	*	*	9
Ko et al. (24)	*	*	*	*	**	*	*	*	9
Qiu et al. (25)	*	*	*	*	**	*	*	*	9
Gong et al. (26)	*	*	*	/	**	/	*	*	7
Xue et al. (27)	*	*	*	*	**	*	*	*	9
Fang et al. (28)	*	*	*	*	**	*	*	*	9
Li et al. (29)	*	*	*	*	**	*	*	*	9
Liu et al. (30)	*	*	*	*	**	*	*	*	9
Liu et al. (31)	*	*	*	*	**	*	*	*	9
Wang et al. (19)	*	*	*	*	*	*	*	*	8
Zong et al. (32)	*	*	*	*	**	*	*	*	9
Renati et al. (33)	*	*	*	*	*	*	*	*	8
Wang et al. (34)	*	*	*	*	**	*	*	*	9
Zhou et al. (35)	*	*	*	/	*	*	*	*	7
Helgason et al. (17)	*	*	*	*	**	*	*	*	9
Tong et al. (36)	*	*	*	*	**	*	*	*	9
Zhang et al. (11)	*	*	*	*	*	*	*	*	8
Chen et al. (37)	*	*	*	*	**	*	*	*	9
Li et al. (38)	*	*	*	*	*	*	*	*	8
Yang et al. (39)	*	*	*	*	**	*	*	*	9
Chen et al. (40)	*	*	*	*	**	*	*	*	9
Li et al. (41)	*	*	*	*	**	*	*	*	9
Yang et al. (42)	*	*	*	/	**	*	*	*	8
Li et al. (43)	*	*	*	*	*	*	*	*	8
Xiao et al. (44)	*	*	*	*	*	*	*	*	8
Xu et al. (45)	*	*	*	*	**	*	*	*	9
Chen et al. (46)	*	*	*	*	**	*	*	*	9
Guan et al. (47)	*	*	*	*	**	*	*	*	9
Xu et al. (18)	*	*	*	*	*	*	*	*	8
Xu et al. (48)	*	*	*	*	**	*	*	*	9
Lei et al. (49)	*	*	*	*	**	*	*	*	8
Li et al. (50)	*	*	*	*	**	*	*	*	9
Abdulwahab et al. (10)	*	*	*	*	**	*	*	*	9
Cai et al. (51)	*	·*	*	*	**	*	*	*	9
Jiang et al. (52)	*	*	*	*	**	*	*	*	9
Li et al. (53)	*	*	*	*	*	*	*	*	8
Liu et al. (54)	*	*	*	*	*	*	*	*	8
Xu et al. (55)	*	*	*	*	**	*	*	*	9
Zhang et al. (56)	*	*	*	*	**	*	*	*	9
Chen et al. (57)	*	*	*	*	**	*	*	*	9
Li et al. (58)	*	*	*	*	**	*	*	*	9
Wang et al. (59)	*	*	*	*	**	*	*	*	9
Zhao et al. (60)	*	*	*	*	*	*	*	*	8

## 4 Meta-analysis results

### 4.1 Incidence of AKI

**Figure 2:**
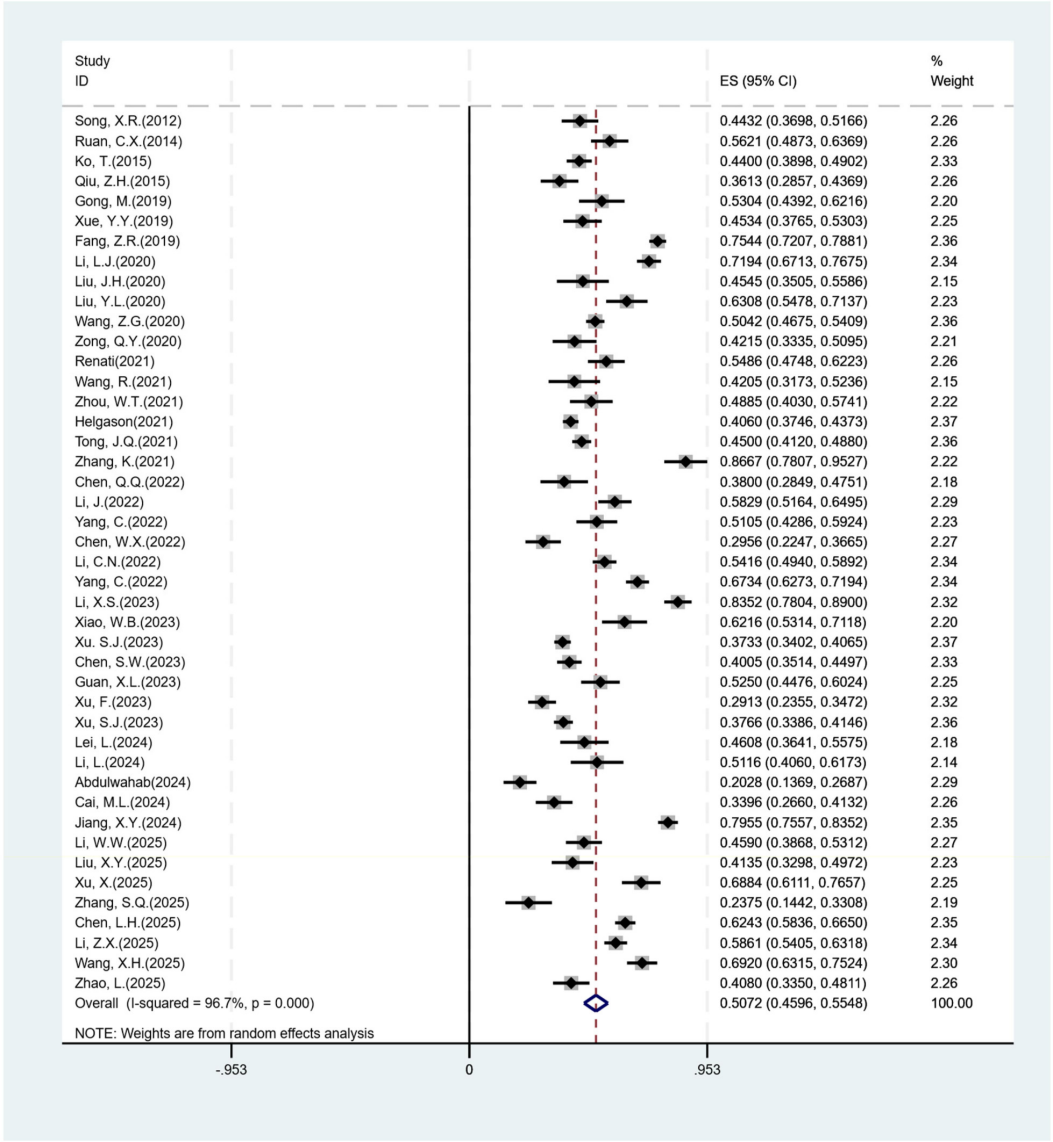
Forest plot of the meta-analysis of incidence of AKI. Squares represent the effect estimates of individual studies, with square size proportional to study weight; horizontal lines indicate 95% confidence intervals; diamonds represent pooled effect estimates.

Forty-four studies reported the incidence of acute kidney injury (AKI) following surgery for acute Stanford Type A aortic dissection. Due to the heterogeneity observed among the 44 included studies (*I*² = 96.7%, *P* = 0.001), a random-effects model was selected to pool the effect sizes. The results (Figure 2) of the meta-analysis indicated that the incidence of AKI following surgery for acute Stanford Type A aortic dissection was 50.72% [95% CI: (45.96%–55.48%)].

### 4.2 Sensitivity analysis and publication bias

A sensitivity analysis was conducted by sequentially excluding each study to assess its impact on the overall pooled estimate. The results (Supplementary Figure S1) indicate that the results were robust. Potential publication bias was evaluated using funnel plots and Egger's test. The funnel plot (Supplementary Figure S2) results demonstrated a largely uniform and symmetrical distribution of data points, while Egger's test (Supplementary Figure S3) indicated minimal publication bias (*t* = −0.73, *P* = 0.472).

### 4.3 Subgroup analysis

**Figure 3:**
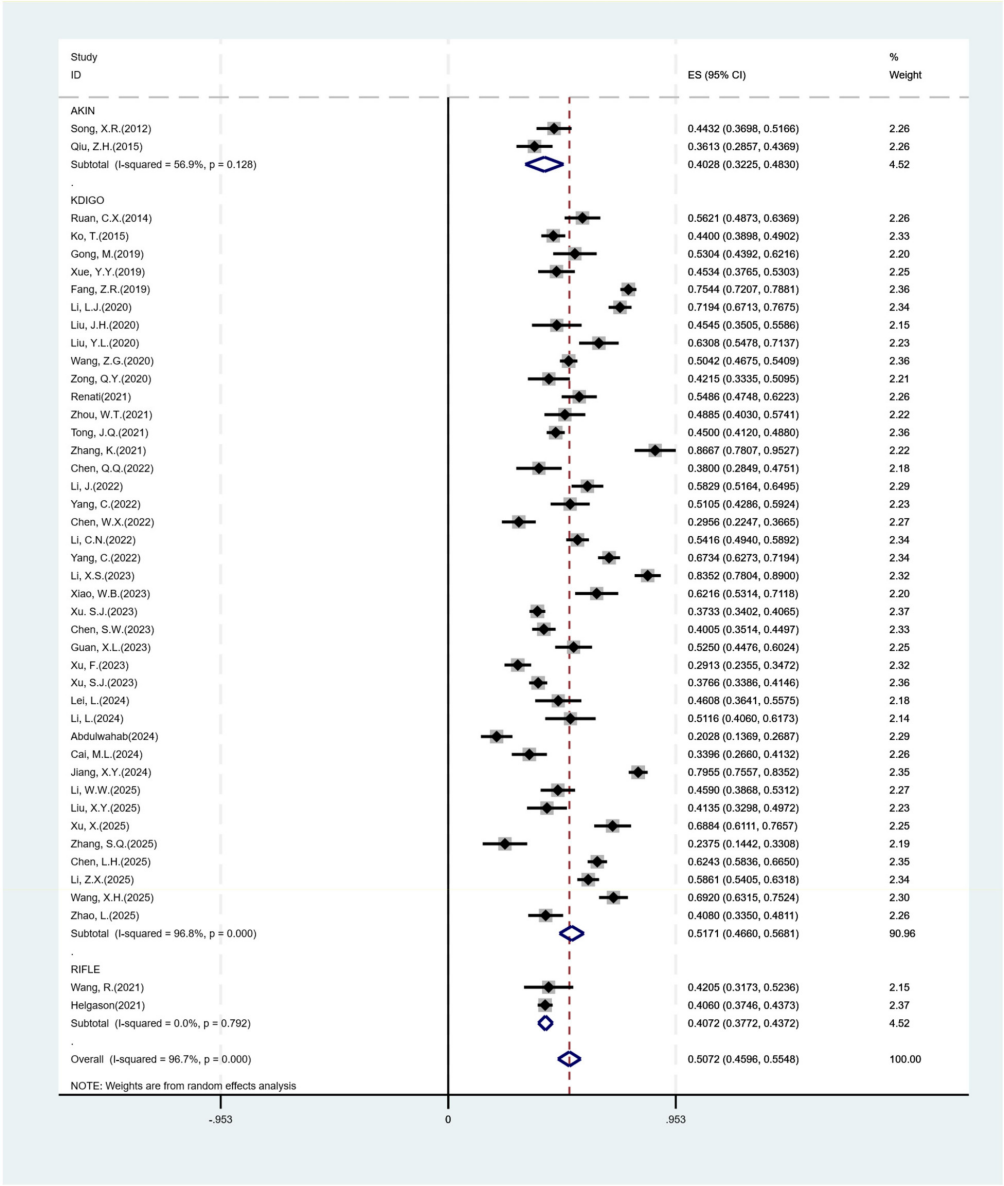
Forest plot of the meta-analysis of incidence of AKI based on diagnostic criteria. Squares represent the effect estimates of individual studies, with square size proportional to study weight; horizontal lines indicate 95% confidence intervals; diamonds represent pooled effect estimates.

**Figure 4:**
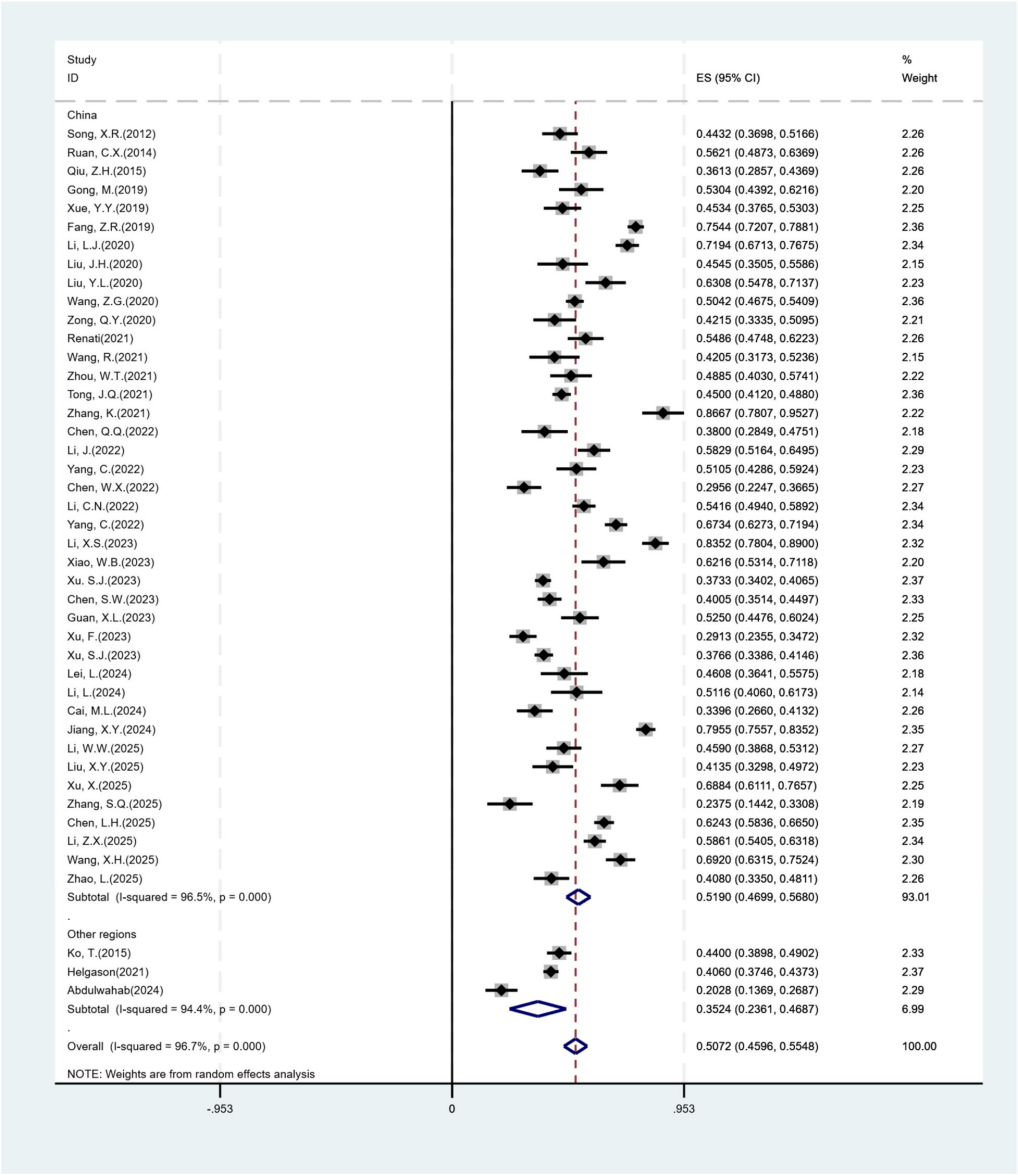
Forest plot of the meta-analysis of incidence of AKI based on regions. Squares represent the effect estimates of individual studies, with square size proportional to study weight; horizontal lines indicate 95% confidence intervals; diamonds represent pooled effect estimates.

Subgroup analyses of the incidence of AKI were conducted based on diagnostic criteria and regions where the study was conducted. The results (Figure 3) indicated that the incidence rate observed using the KDIGO diagnostic criteria [51.71%, 95% CI: (46.60%, 56.81%)] was significantly higher than that observed using the AKIN criteria [40.28%, 95% CI: (32.25%, 48.30%)] or the RIFLE criteria [40.72%, 95% CI: (45.96%, 55.48%)], suggesting that the KDIGO criteria may be more sensitive in diagnosing AKI. Stratification by study region revealed that the reported incidence of AKI (Figure 4) in studies conducted in China [51.90%, 95% CI: (46.99%, 56.80%)] was higher than that in studies conducted in regions outside of China [35.24%, 95% CI: (26.61%, 46.87%)].

### 4.4 Risk factors results

#### 4.4.1 Prolonged cardiopulmonary bypass

**Figure 5:**
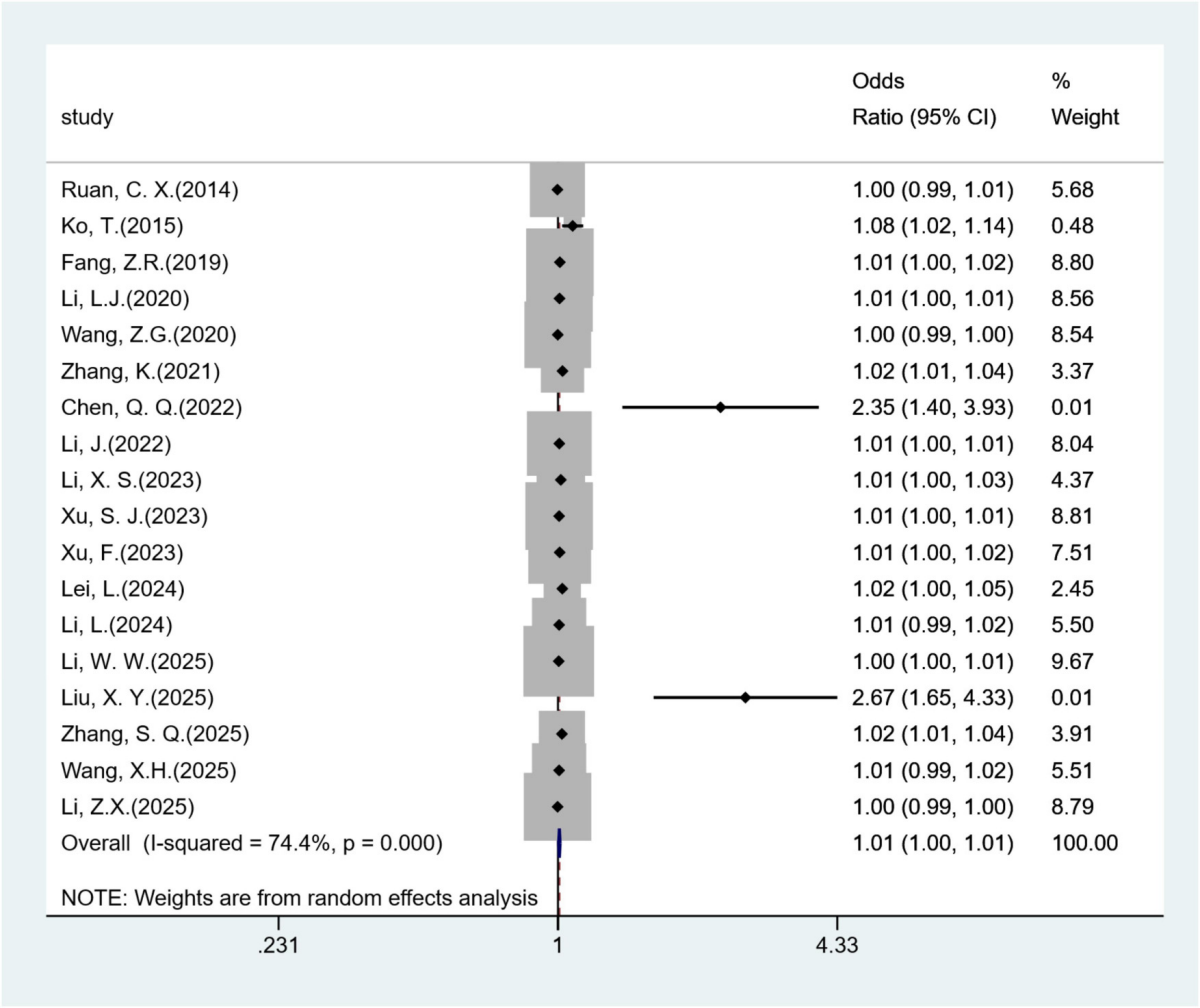
Forest plot of the meta-analysis of prolonged cardiopulmonary bypass. Squares represent the effect estimates of individual studies, with square size proportional to study weight; horizontal lines indicate 95% confidence intervals; diamonds represent pooled effect estimates.

A total of 18 articles reported prolonged cardiopulmonary bypass (CBP). Heterogeneity was assessed using a random-effects model (*I*^2^ = 74.4%, *P* = 0.001). The pooled analysis (Figure 5) indicated that CBP duration may be associated with an increased risk of AKI following surgery for TAAD [OR = 1.01, 95% CI: (1.00, 1.01)]. Due to the significant heterogeneity, a sensitivity analysis was performed by sequentially excluding individual studies. The results (Supplementary Figure S4) demonstrated that this indicator remained robust.

#### 4.4.2 BMI

**Figure 6:**
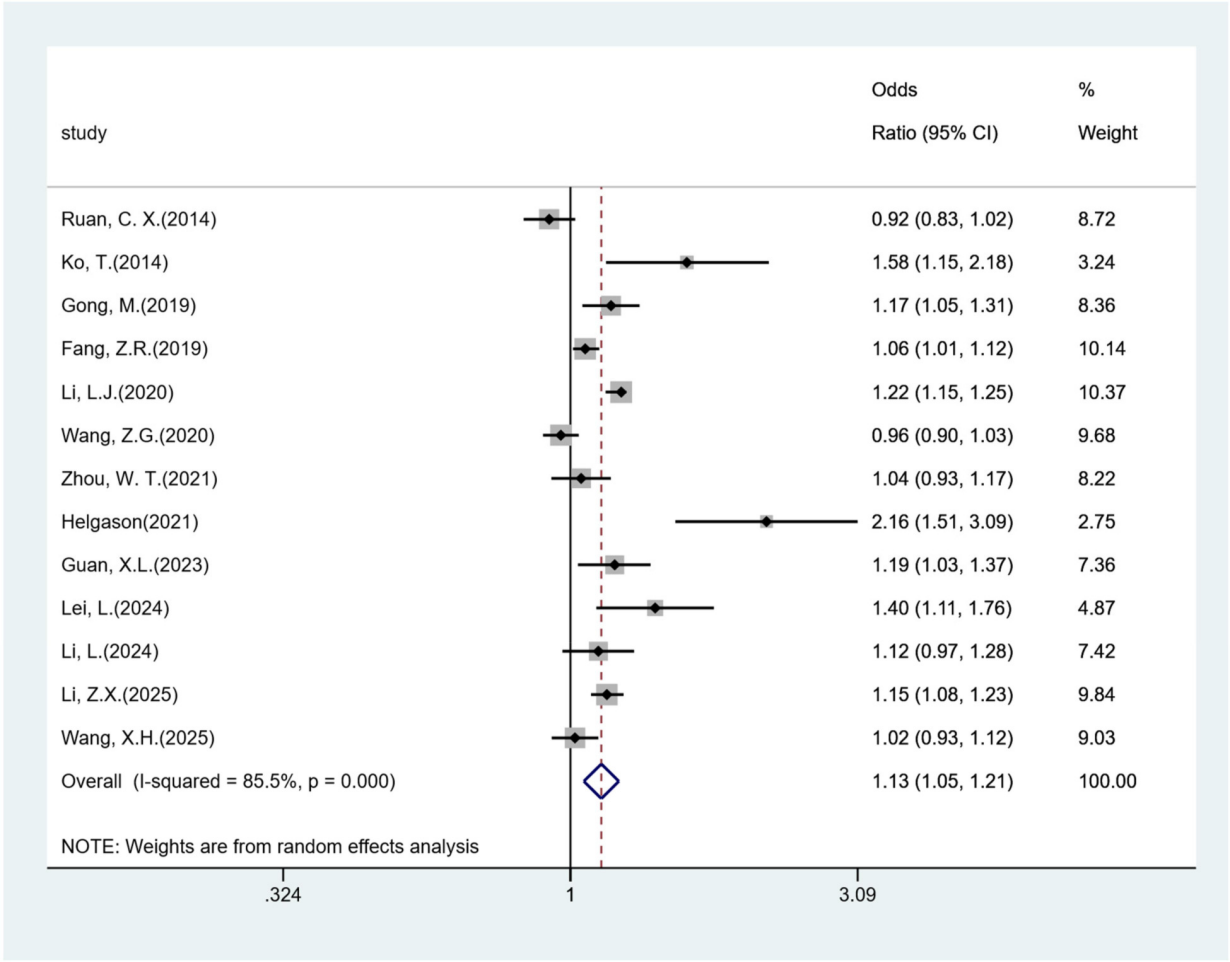
Forest plot of the meta-analysis of BMI. Squares represent the effect estimates of individual studies, with square size proportional to study weight; horizontal lines indicate 95% confidence intervals; diamonds represent pooled effect estimates.

A total of 13 articles mentioned BMI; a random-effects model was employed for the heterogeneity test (*I*^2^ = 85.5%, *P* = 0.001). The pooled analysis (Figure 6) indicated that BMI may be associated with an increased risk of AKI following surgery for Stanford Type A aortic dissection [OR = 1.13, 95% CI: (1.05, 1.21)]. Given the significant heterogeneity, a sensitivity analysis was conducted by sequentially excluding individual studies. The results (Supplementary Figure S5) demonstrated that the finding remained robust.

#### 4.4.3 History of hypertension

**Figure 7:**
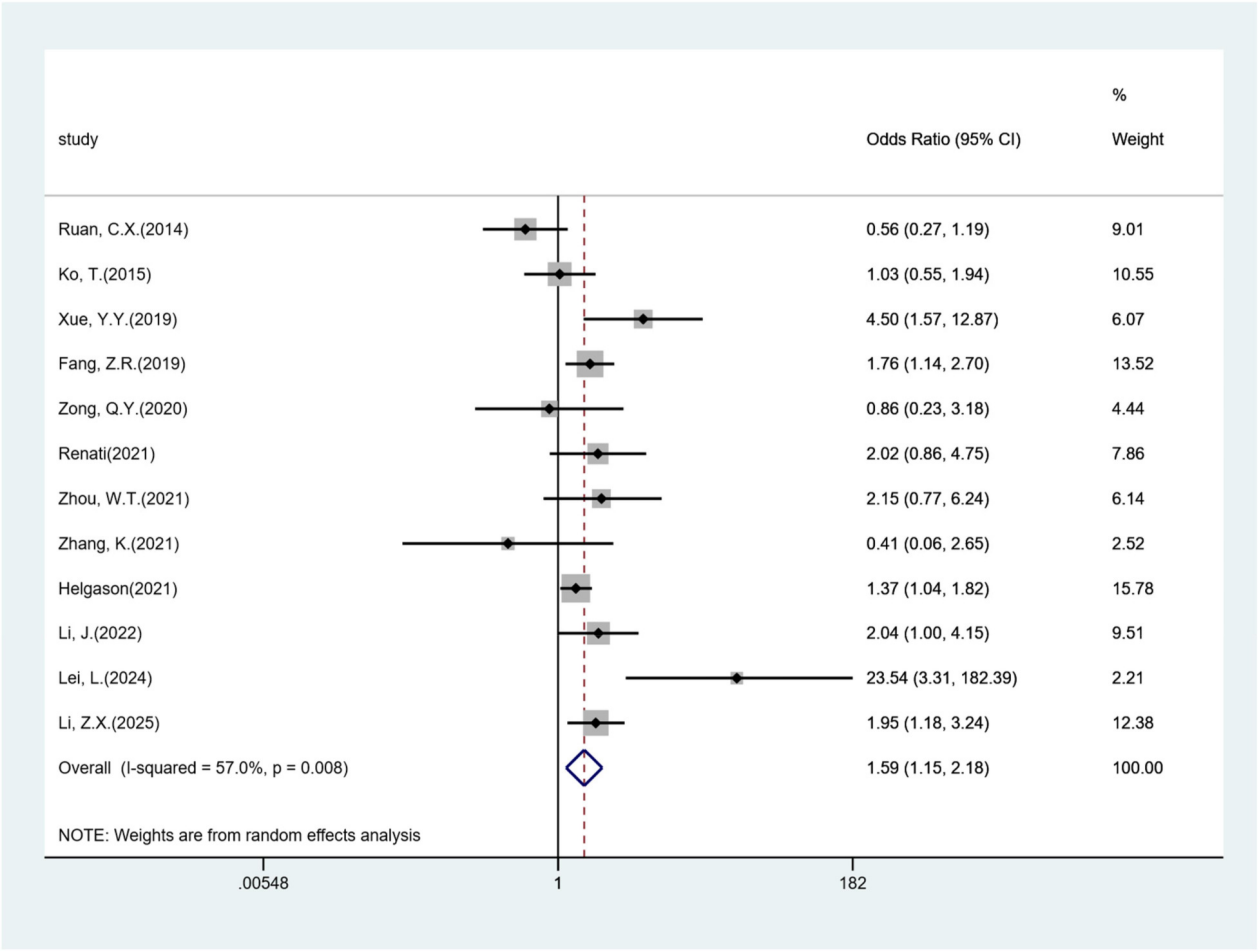
Forest plot of the meta-analysis of history of hypertension. Squares represent the effect estimates of individual studies, with square size proportional to study weight; horizontal lines indicate 95% confidence intervals; diamonds represent pooled effect estimates.

A total of 12 articles mentioned a history of hypertension; heterogeneity testing (*I*^2^ = 57.0%, *P* = 0.008) was conducted using a random-effects model. The pooled analysis (Figure 7) indicated that a history of hypertension may be associated with an increased risk of postoperative AKI in patients with TAAD [OR = 1.59, 95% CI: (1.15, 2.18)]. Given the significant heterogeneity, a sensitivity analysis was performed by sequentially excluding individual studies. The results (Supplementary Figure S6) demonstrated that this finding remained robust.

#### 4.4.4 Preoperative serum creatinine

**Figure 8:**
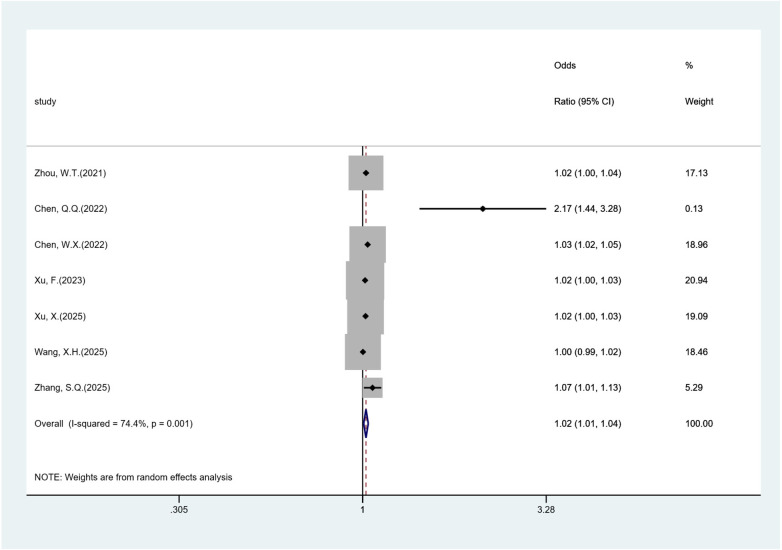
Forest plot of the meta-analysis of preoperative serum creatinine. Squares represent the effect estimates of individual studies, with square size proportional to study weight; horizontal lines indicate 95% confidence intervals; diamonds represent pooled effect estimates.

A total of seven articles mentioned preoperative serum creatinine. Due to significant heterogeneity (*I*^2^ = 74.4%, *P* = 0.001), a random-effects model was employed. The pooled analysis (Figure 8) indicated that preoperative serum creatinine may be associated with an increased risk of postoperative AKI in patients with TAAD [OR = 1.02, 95% CI: (1.01, 1.04)]. Given the significant heterogeneity, a sensitivity analysis was conducted by sequentially excluding individual studies. The results (Supplementary Figure S7) demonstrated that this indicator remained robust.

#### 4.4.5 Age

**Figure 9:**
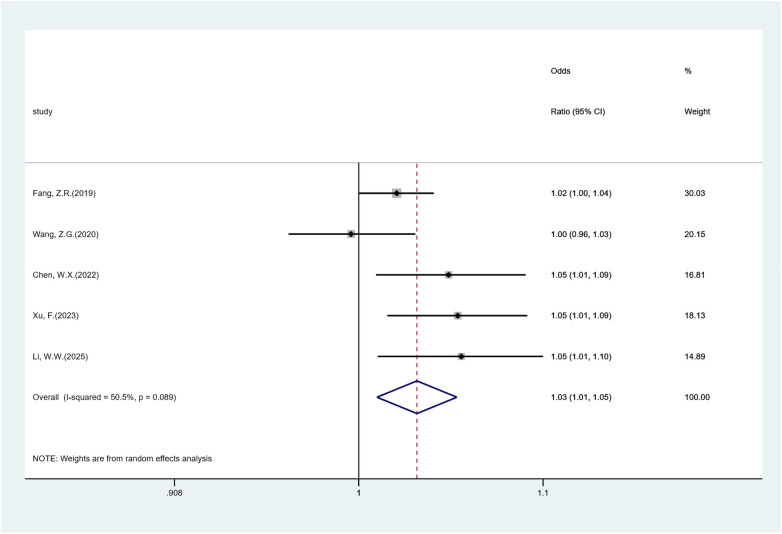
Forest plot of the meta-analysis of age (per 1 year). Squares represent the effect estimates of individual studies, with square size proportional to study weight; horizontal lines indicate 95% confidence intervals; diamonds represent pooled effect estimates.

**Figure 10:**
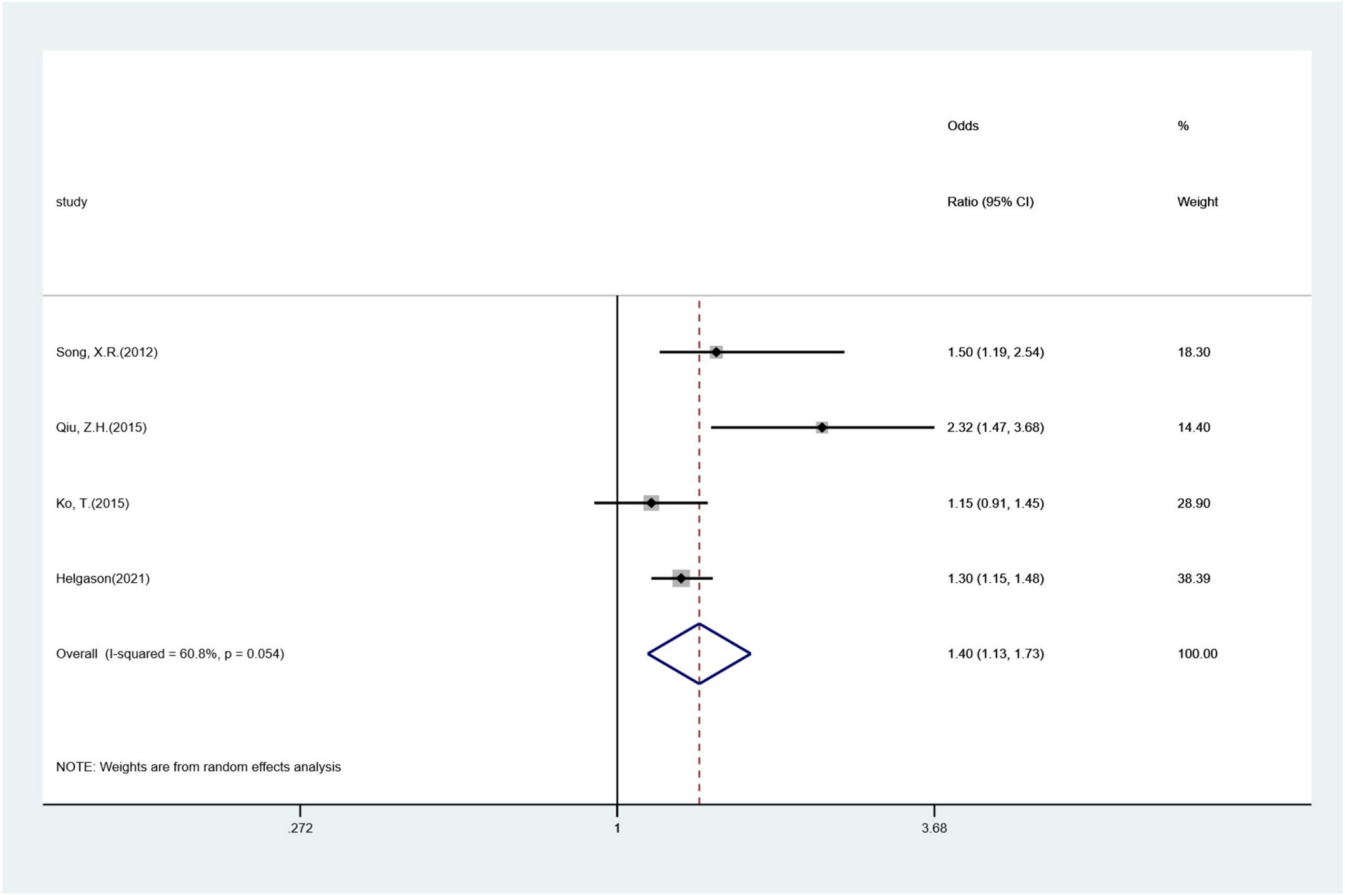
Forest plot of the meta-analysis of age (per 10 years). Squares represent the effect estimates of individual studies, with square size proportional to study weight; horizontal lines indicate 95% confidence intervals; diamonds represent pooled effect estimates.

A total of nine studies mentioned the age. Recognizing that the definition of age varied across different studies, a subgroup analysis was conducted. Five studies defined age in terms of a 1-year increment, while four studies defined it in terms of a 10-year increment. In the “1-year increment” subgroup, due to significant heterogeneity (*I*^2^ = 50.5%, *P* = 0.089), a random-effects model was employed. The pooled analysis (Figure 9) indicated that each 1-year increase in age may be associated with an increased risk of postoperative AKI in patients with TAAD [OR = 1.03, 95% CI: (1.01, 1.05)]. Given the significant heterogeneity, a sensitivity analysis was performed by sequentially excluding individual studies. The results (Supplementary Figure S8) demonstrated that this association remained robust. In the “10-year increment” subgroup, due to significant heterogeneity (*I*^2^ = 60.8%, *P* = 0.054), a random-effects model was employed. The pooled analysis (Figure 10) suggested that each 10-year increase in age may be associated with an increased risk of postoperative AKI in patients with TAAD [OR = 1.40, 95% CI: (1.13, 1.73)]. Due to the significant heterogeneity, a sensitivity analysis was conducted by sequentially excluding individual studies. The results (Supplementary Figure S10) indicated that this association remained robust.

#### 4.4.6 Prolonged duration of surgery

**Figure 11:**
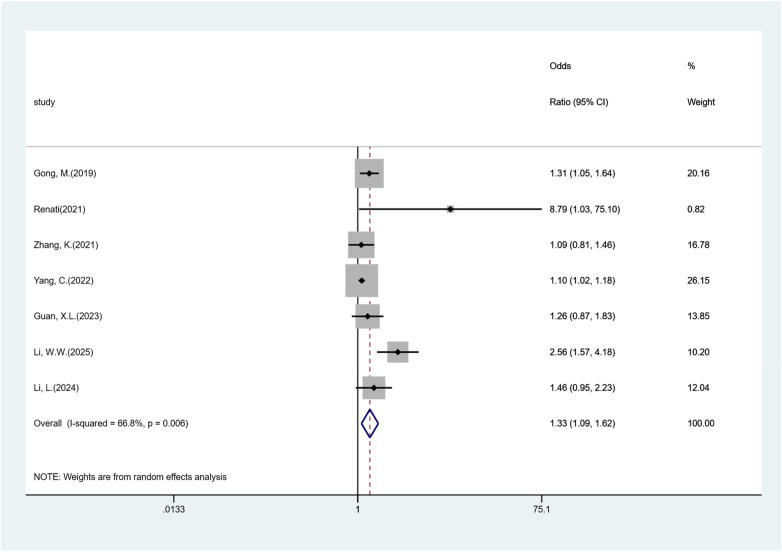
Forest plot of the meta-analysis of prolonged duration of surgery. Squares represent the effect estimates of individual studies, with square size proportional to study weight; horizontal lines indicate 95% confidence intervals; diamonds represent pooled effect estimates.

A total of seven articles reported the prolonged duration of surgery. Heterogeneity testing (*I*^2^ = 66.8%, *P* = 0.006) was conducted using a random-effects model. The pooled analysis (Figure 11) indicated that prolonged surgery duration may be associated with an increased risk of postoperative AKI in patients with TAAD [OR = 1.33, 95% CI: (1.09, 1.62)]. Given the significant heterogeneity, a sensitivity analysis was performed by sequentially excluding individual studies. The results (Supplementary Figure S10) demonstrated that this finding remained robust.

#### 4.4.7 Preoperative leukocyte count

**Figure 12:**
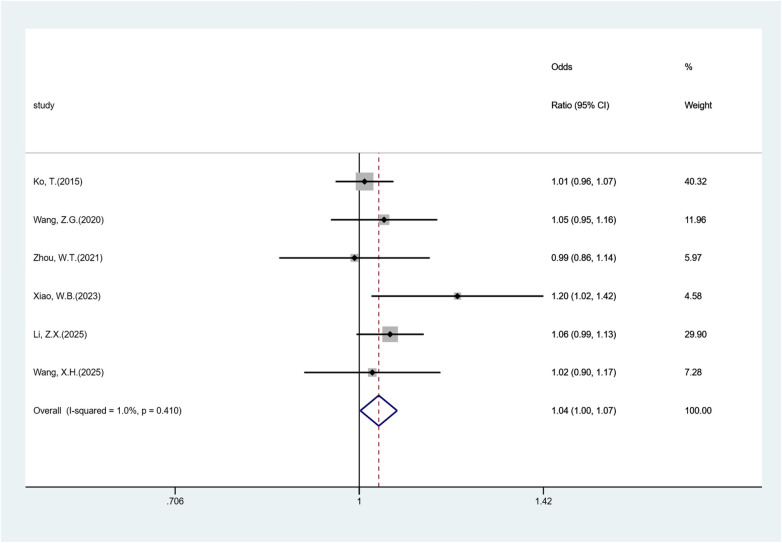
Forest plot of the meta-analysis of preoperative leukocyte counts. Squares represent the effect estimates of individual studies, with square size proportional to study weight; horizontal lines indicate 95% confidence intervals; diamonds represent pooled effect estimates.

A total of six articles reported the preoperative leukocyte counts. Heterogeneity testing (*I*^2^ = 1.0%, *P* = 0.410) was performed using a fixed-effects model. The pooled analysis (Figure 12) indicated that preoperative leukocyte counts may be associated with an increased risk of postoperative AKI in patients with TAAD [OR = 1.04, 95% CI: (1.00, 1.07)].

#### 4.4.8 Perioperative red blood cell transfusion volume

**Figure 13:**
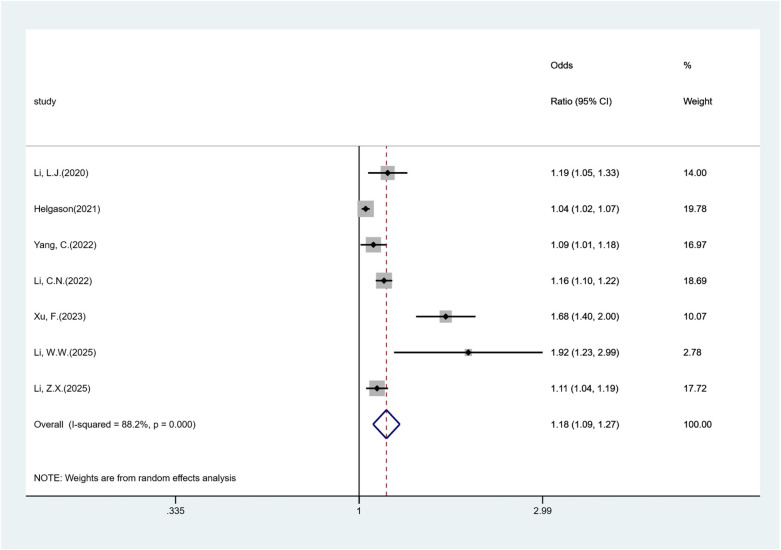
Forest plot of the meta-analysis of the volume of perioperative red blood cell transfusion. Squares represent the effect estimates of individual studies, with square size proportional to study weight; horizontal lines indicate 95% confidence intervals; diamonds represent pooled effect estimates.

A total of seven articles reported the volume of perioperative red blood cell transfusion; heterogeneity testing (*I*^2^ = 88.2%, *P* = 0.001) was conducted using a random-effects model. The pooled analysis (Figure 13) indicated that the volume of perioperative red blood cell transfusion may be associated with an increased risk of postoperative AKI in patients with Stanford Type A aortic dissection [OR = 1.18, 95% CI: (1.09, 1.27)]. Given the significant heterogeneity, a sensitivity analysis was performed by sequentially excluding individual studies. The results (Supplementary Figure S11) demonstrated that this indicator remained robust.

#### 4.4.9 Gender

**Figure 14:**
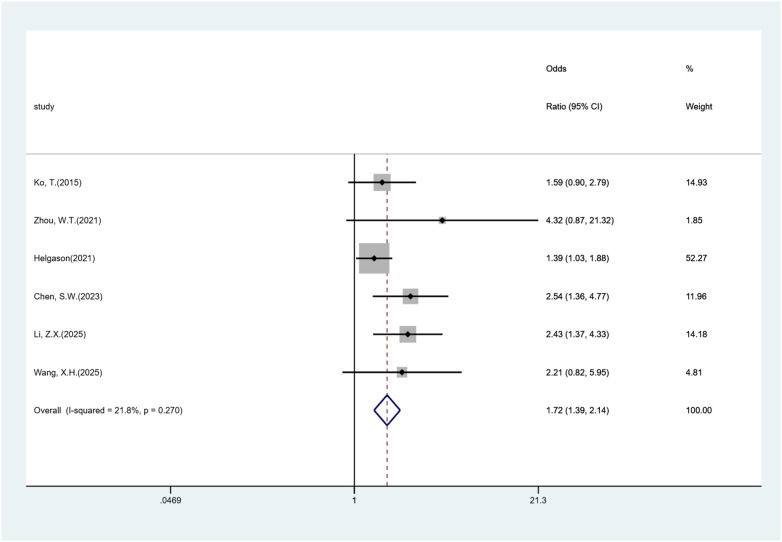
Forest plot of the meta-analysis of male. Squares represent the effect estimates of individual studies, with square size proportional to study weight; horizontal lines indicate 95% confidence intervals; diamonds represent pooled effect estimates.

A total of seven articles addressed gender, treating male sex as a potential influencing factor; heterogeneity testing (*I*^2^ = 21.8%, *P* = 0.270) was conducted using a fixed-effects model. The pooled analysis (Figure 14) indicated that male sex may be associated with an increased risk of AKI following surgery for TAAD [OR = 1.72, 95% CI: (1.39, 2.14)].

#### 4.4.10 Deep hypothermic circulatory arrest duration

**Figure 15:**
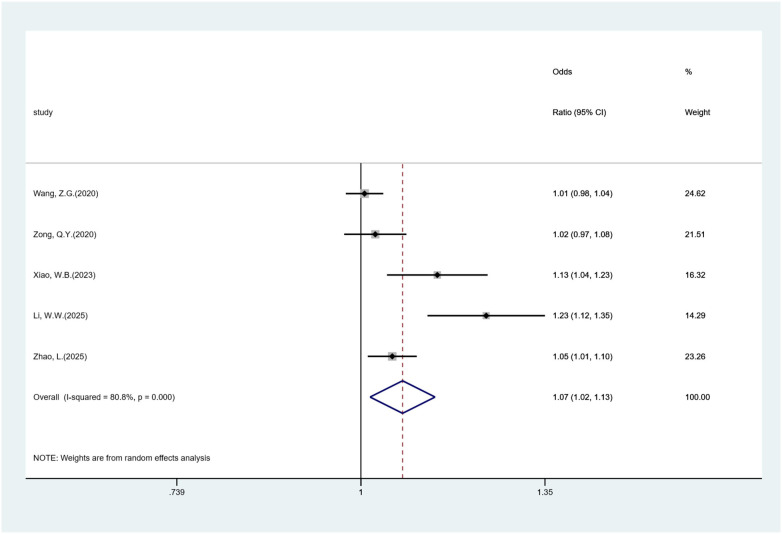
Forest plot of the meta-analysis of the duration of deep hypothermic circulatory arrest. Squares represent the effect estimates of individual studies, with square size proportional to study weight; horizontal lines indicate 95% confidence intervals; diamonds represent pooled effect estimates.

A total of five articles addressed the duration of deep hypothermic circulatory arrest. Heterogeneity testing (*I*^2^ = 80.8%, *P* = 0.001) was conducted using a random-effects model. The pooled analysis (Figure 15) indicated that the duration of deep hypothermic circulatory arrest may be associated with an increased risk of postoperative AKI in patients with TAAD [OR = 1.07, 95% CI: (1.02, 1.13)]. Given the significant heterogeneity, a sensitivity analysis was performed by sequentially excluding individual studies. The results (Supplementary Figure S12) demonstrated that this indicator remained robust.

#### 4.4.11 Duration of perioperative mechanical ventilation

**Figure 16:**
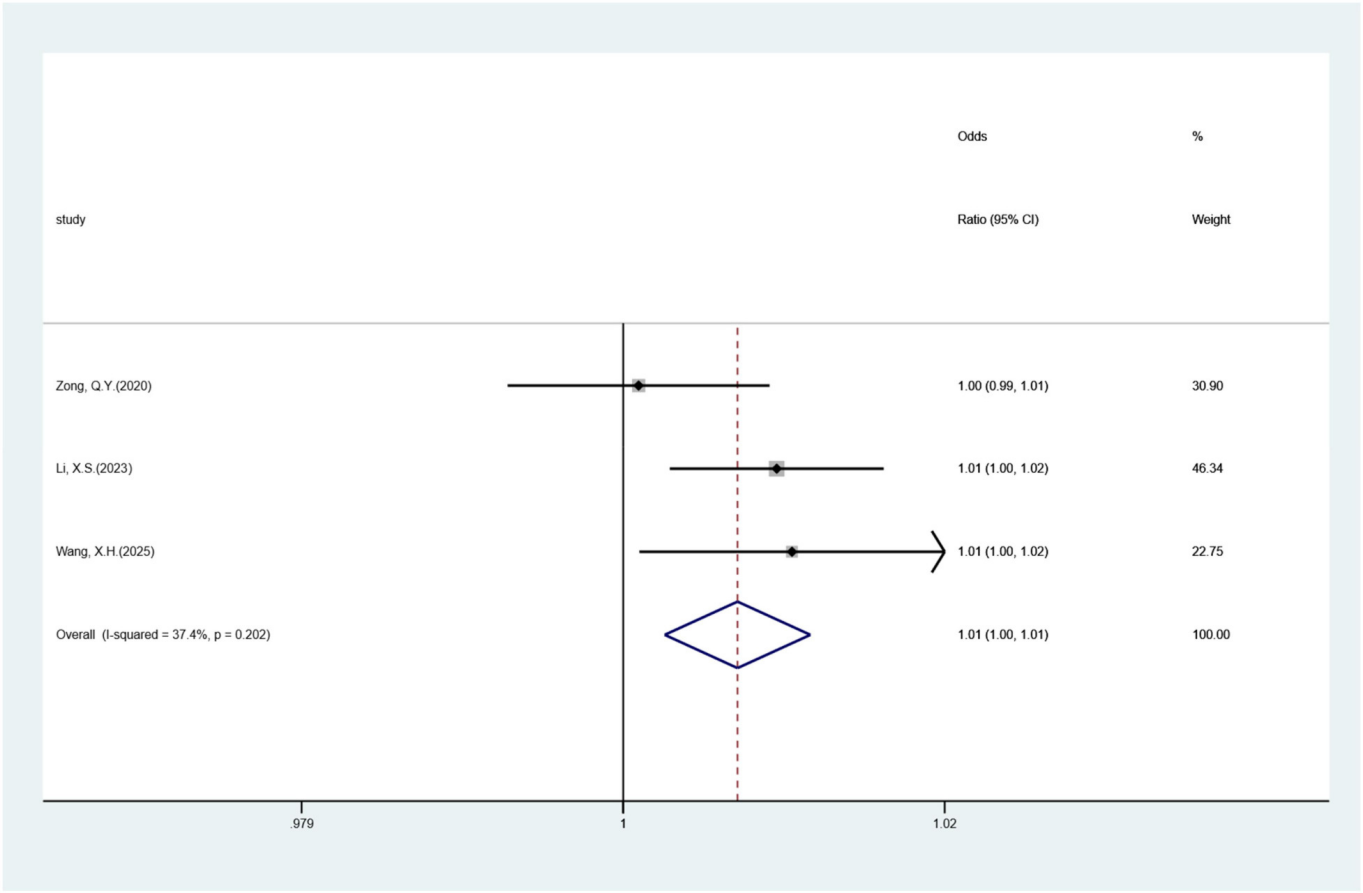
Forest plot of the meta-analysis of the duration of perioperative mechanical ventilation. Squares represent the effect estimates of individual studies, with square size proportional to study weight; horizontal lines indicate 95% confidence intervals; diamonds represent pooled effect estimates.

A total of three articles reported the duration of perioperative mechanical ventilation; heterogeneity testing (*I*^2^ = 37.4%, *P* = 0.202) was performed using a fixed-effects model. The pooled analysis (Figure 16) indicated that mechanical ventilation duration may be associated with an increased risk of AKI following surgery for TAAD [OR = 1.01, 95% CI: (1.00, 1.01)].

#### 4.4.12 Preoperative dissection involving the renal artery

**Figure 17:**
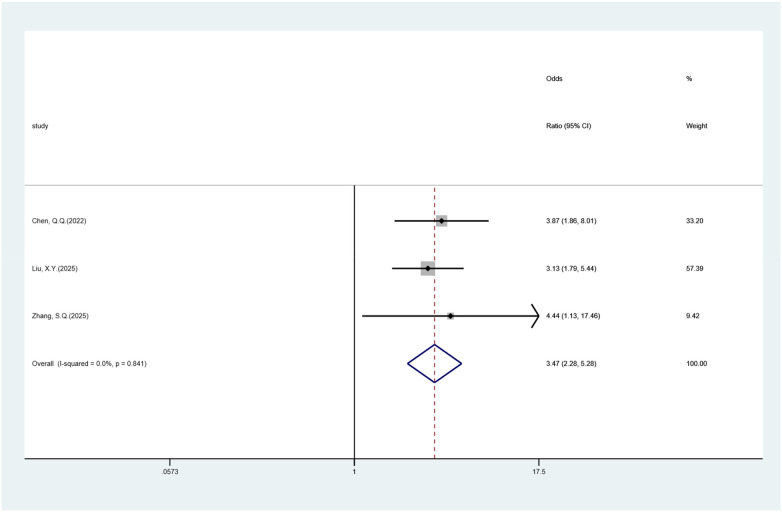
Forest plot of the meta-analysis of preoperative dissection involving the renal artery. Squares represent the effect estimates of individual studies, with square size proportional to study weight; horizontal lines indicate 95% confidence intervals; diamonds represent pooled effect estimates.

A total of three articles reported preoperative dissection involving the renal artery; heterogeneity testing (*I*^2^ = 0.0%, *P* = 0.841) was performed using a fixed-effects model. The pooled analysis (Figure 17) indicated that renal artery involvement may be associated with an increased risk of postoperative AKI in patients with TAAD [OR = 3.47, 95% CI: (2.28, 5.28)].

#### 4.4.13 Preoperative lactate level

**Figure 18:**
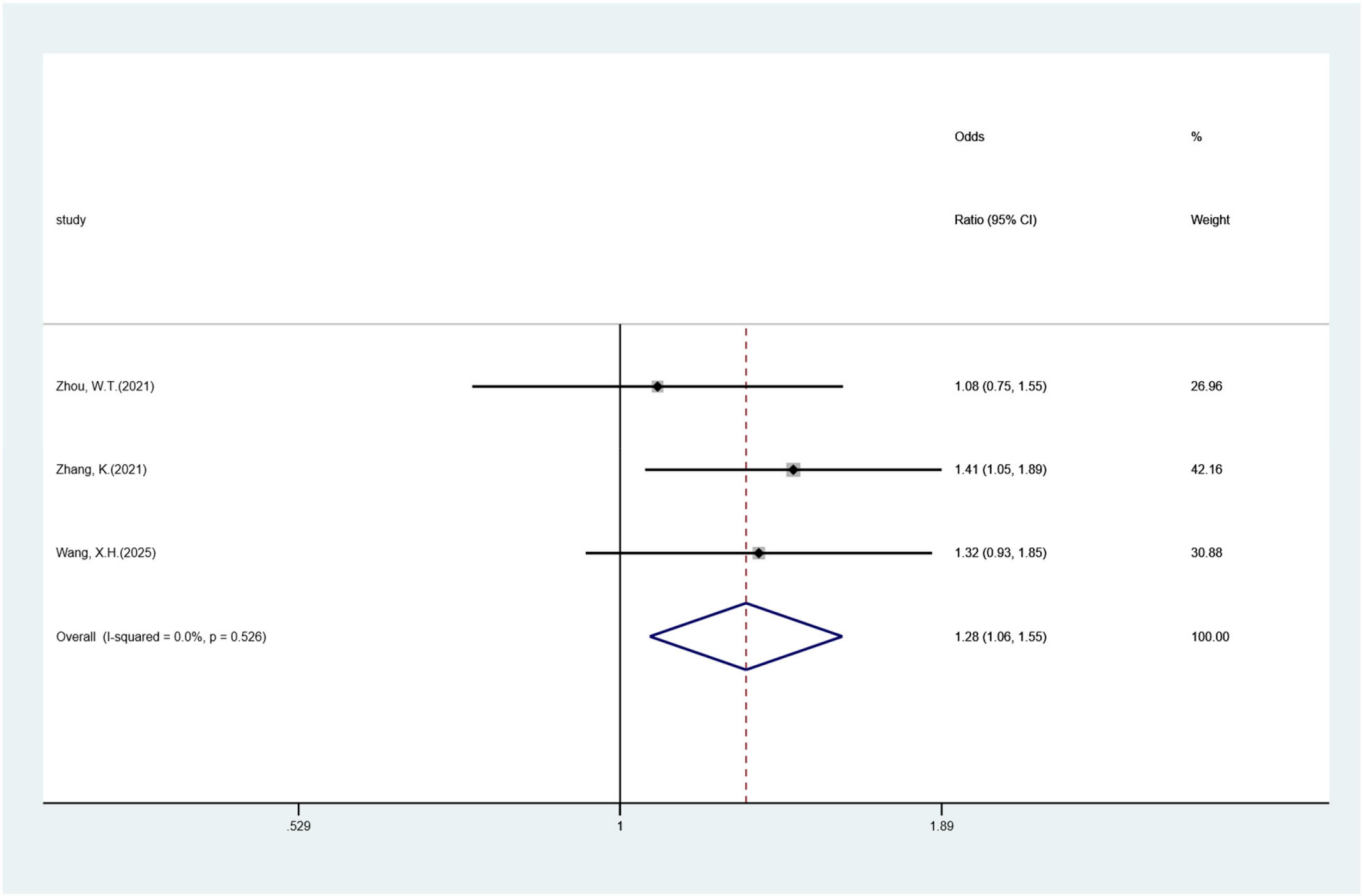
Forest plot of the meta-analysis of the preoperative lactate levels. Squares represent the effect estimates of individual studies, with square size proportional to study weight; horizontal lines indicate 95% confidence intervals; diamonds represent pooled effect estimates.

A total of three articles reported the preoperative lactate levels; heterogeneity testing (*I*^2^ = 0.0%, *P* = 0.526) was performed using a fixed-effects model. The pooled analysis (Figure 18) indicated that preoperative lactate may be associated with an increased risk of postoperative AKI in patients with TAAD [OR = 1.28, 95% CI: (1.06, 1.55)].

#### 4.4.14 Publication bias

This study employed funnel plots and Egger's test to detect publication bias. The results (Supplementary Figure S13–S40) indicated that publication bias may exist regarding prolonged cardiopulmonary bypass (*P* = 0.001), preoperative serum creatinine (*P* = 0.044), duration of surgery (*P* = 0.026), the volume of red blood cell transfusion (*P* = 0.012), male (*P* = 0.040), and duration of deep hypothermic circulatory arrest (*P* = 0.028).

#### 4.4.15 Trim-and-fill results

Due to publication bias associated with prolonged cardiopulmonary bypass (CPB), preoperative serum creatinine levels, duration of surgery, volume of red blood cell transfusion, male and duration of deep hypothermic circulatory arrest, the trim-and-fill method was employed to further assess the robustness of the results. The findings (Supplementary Figure S41–S46) indicate that, even in the presence of publication bias, the conclusions remain robust.

## 5 Discussion

### 5.1 The incidence of AKI after Stanford type A aortic dissection is high

The meta-analysis results of this study showed that the incidence of AKI after Stanford type A aortic dissection was as high as 50.49%, which was significantly higher than the incidence of AKI after heart valve replacement (61) and coronary artery bypass surgery (62). Type A aortic dissection surgery usually requires deep hypothermic circulatory arrest and selective cerebral perfusion, involving the replacement of the aortic arch and even the thoracic and abdominal aorta. This high incidence of postoperative AKI may be related to the long-term hypoperfusion or even nonperfusion of the kidneys during the operation (17, 63). Low pressure non-pulsatile perfusion, rapid temperature changes, blood dilution and other factors during cardiopulmonary bypass may also lead to insufficient kidney perfusion. Blood exposure in the cardiopulmonary bypass circuit leads to the activation of inflammatory factors and oxidative stress (64). Surgical damage to tissues and mechanical destruction of red blood cells may further increase the burden on kidney function, leading to a decrease in glomerular filtration rate and, in severe cases, kidney damage (65). Postoperative high-dose use of vasoactive drugs, excessive or insufficient volume overload, and possible low cardiac output syndrome may all create a pathophysiological environment for the occurrence of postoperative AKI (66, 67). Subgroup analysis of this study showed that the incidence of AKI was higher in studies diagnosed using the KDIGO criteria, which may be related to the fact that the KDIGO criteria are more sensitive to small changes in serum creatinine, and can identify AKI patients earlier and more comprehensively (68). Therefore, it is recommended that the KDIGO criteria be used to diagnose AKI in the future to achieve early detection and early treatment. At the same time, we found that the incidence in Chinese studies was higher than that in non-Chinese regions, and there are several reasons for this difference. This may be related to the fact that Chinese TAAD patients are more severely ill at the time of diagnosis, and have a higher prevalence of underlying diseases such as hypertension and diabetes, but the situation of cardiovascular risk factor control in China is not good (69). Although the annual surgical volume of large cardiovascular centers in China is large, the perioperative details such as extracorporeal circulation management and blood transfusion strategy are not well managed (70).

### 5.2 Preoperative baseline patient status as a risk factor for AKI development

The results of this study indicate that age, high BMI, a history of hypertension, and male are risk factors for postoperative AKI following surgery for TAAD. With advancing age, there is a substantial loss of nephrons, and the glomerular filtration rate declines by approximately 0.80 mL/min/1.73 m² per year (71). Impaired renal tubular function leads to a reduction in renal reserve capacity, rendering the kidneys more susceptible to intraoperative hypotension, ischemia, and toxic insults. Concurrently, as the kidneys age, endothelial dysfunction emerges, resulting in diminished intrarenal hemodynamic regulatory capabilities; consequently, in the presence of volume depletion, shock, or surgical stress, the kidneys become highly prone to developing renal tubular ischemia-reperfusion injury (72). Individuals with a high BMI are frequently characterized by adipose tissue expansion and insulin resistance, leading to the excessive release of free fatty acids and ectopic lipid accumulation. This, in turn, triggers lipotoxicity, mitochondrial dysfunction, and persistent oxidative stress and inflammation (73). During the perioperative period, exposure to additional insults, such as ischemia-reperfusion injury, systemic inflammatory activation, and hemodynamic instability, further exacerbates the generation of reactive oxygen species and cellular damage. This heightened vulnerability increases the kidney's susceptibility to injury and significantly elevates the risk of postoperative AKI (74, 75). Chronic hypertension activates the renin-angiotensin-aldosterone system, promoting renal arteriolosclerosis and subsequent renal parenchymal ischemia, which can result in impaired renal function. Relevant studies have demonstrated that renal cortical perfusion is lower in patients with hypertension compared to the general population (76). Furthermore, this study identified gender as a potential contributing factor to the development of postoperative AKI following TAAD surgery. This phenomenon may be associated with estrogen levels. A multicenter retrospective cohort study revealed that the incidence of postoperative AKI is lower in younger women compared to older women and men across all age groups; however, the incidence of AKI gradually increases with advancing age (77). An animal study has further revealed that, following renal ischemia, male mice exhibit a significant upregulation in the expression of proximal tubule injury markers and pro-inflammatory factors, whereas female mice demonstrate greater resilience and more subtle transcriptomic changes (78). These findings suggest that the anti-inflammatory and antioxidant effects of estrogen—along with the activation of apoptotic pathways by testosterone—may constitute the core mechanisms underlying these sex-based differences. Consequently, for individuals at high risk of developing postoperative AKI, preventive measures should be implemented early, with particular attention paid to assessing their baseline health status. Specifically, for patients with long-standing comorbidities such as hypertension and high BMI, preoperative blood pressure control and weight management should be optimized to mitigate the risk of postoperative AKI.

### 5.3 Preoperative renal status as a risk factor for AKI

This study identified elevated preoperative serum creatinine levels and renal artery involvement as risk factors for postoperative AKI after TAAD surgery. Meta-analysis of preoperative serum creatinine showed a statistically significant but weak association with postoperative AKI [OR = 1.02, 95% CI: (1.01, 1.04)]. Elevated preoperative serum creatinine reflects insufficient renal functional reserve prior to surgery. Because TAAD surgery usually requires cardiopulmonary bypass, kidneys that are already compromised may suffer additional insults from intraoperative hemodynamic fluctuations and hypothermic circulatory arrest, such as hypoperfusion and ischemia-reperfusion injury, making AKI more likely to occur (79, 80). In contrast, renal artery involvement is a direct manifestation of anatomical and hemodynamic impairment of renal perfusion. When the dissection extends into the renal arteries, formation of a false lumen disrupts renal blood flow, leading to an imbalance between oxygen supply and demand within renal cells and resulting in necrosis and apoptosis of specific glomeruli, renal tubules, and interstitial cells. Consequently, the kidney's capacity to process metabolic waste diminishes, ultimately leading to impaired renal function (81). These findings underscore the critical importance of early identification of renal perfusion status and assessment of baseline renal function.

During our analysis, we also identified other preoperative renal indicators, including chronic kidney disease (CKD), estimated glomerular filtration rate (eGFR), and serum cystatin C. However, each of these was reported in only two studies, and their definitions varied, precluding quantitative synthesis. Collectively, these results indicate that impaired preoperative renal function is associated with an increased risk of postoperative AKI, although the effect size of serum creatinine alone is clinically modest. This suggests that while serum creatinine has some predictive value, broader renal indicators, especially CKD, may be stronger predictors of postoperative AKI. The limited number of studies and inconsistent definitions highlight the need for standardized preoperative renal assessment in future research. Clinicians should interpret mild changes in serum creatinine cautiously and consider a comprehensive evaluation of renal function when assessing the risk of postoperative AKI in patients undergoing Stanford type A aortic dissection surgery. We recommend that individualized renal protective strategies be optimized according to each patient's baseline renal status before surgery to reduce the incidence of postoperative AKI.

### 5.4 Surgical trauma as a risk factor for AKI

This study revealed that the duration of cardiopulmonary bypass (CPB), total operative time, and the duration of deep hypothermic circulatory arrest (DHCA) are significantly associated with the risk of AKI. The occurrence of AKI during CPB may be attributed to reduced renal perfusion pressure, the activation of pro-inflammatory factors, direct nephrotoxicity, and hemolysis (82); these factors may all be exacerbated as the duration of CPB and total operative time increase. DHCA, a technique frequently employed during surgery for Type A aortic dissection to address arch pathologies, provides a bloodless surgical field but comes at the cost of compromising systemic organ perfusion; the procedure of hypothermic circulatory arrest impairs the function of vital organs such as the liver and kidneys. Following the resumption of cardiac activity, the kidneys undergo a second hit in the form of ischemia-reperfusion injury (83). Furthermore, a prolonged operative time often entails a correspondingly longer duration of aortic cross-clamping; this extends the period of renal ischemia and hypoxia, thereby intensifying the degree of ischemia-reperfusion injury, which in turn contributes to the development of postoperative AKI. Consequently, optimizing perfusion strategies and implementing real-time monitoring of renal perfusion during surgery may serve as critical intervention points for mitigating AKI and improving patient outcomes.

### 5.5 Perioperative red blood cell transfusion volume and duration of mechanical ventilation are risk factors for AKI

This study also revealed a significant association between the volume of perioperative red blood cell transfusions and the duration of mechanical ventilation with the risk of AKI. Although massive intraoperative red blood cell transfusion aims to correct anemia and maintain oxygen delivery, its potential nephrotoxicity cannot be overlooked. During storage, banked red blood cells release free hemoglobin and iron ions, generating reactive oxygen species that induce lipid peroxidation and mitochondrial dysfunction (84). Furthermore, transfusion-related immunomodulatory effects may suppress host immune surveillance, thereby increasing the risk of infection and indirectly exacerbating renal injury (85). Concurrently, in the perioperative period, patients with Type A aortic dissection often require prolonged mechanical ventilation to support respiration. The use of high positive end-expiratory pressure during mechanical ventilation elevates intrathoracic pressure, impairs right ventricular function, and increases pulmonary vascular resistance; this leads to elevated central venous pressure and reduced renal blood flow, resulting in renal venous congestion and parenchymal stasis. Consequently, reduced capillary blood flow further exacerbates local renal hypoxia (86). Moreover, mechanical ventilation may also increase intra-abdominal pressure, thereby compromising renal microvascular blood flow; in turn, renal edema further elevates intra-abdominal pressure, creating a vicious cycle that intensifies renal dysfunction (87). These findings suggest that, in clinical practice, priority should be given to the use of autologous blood salvage or washed red blood cells when correcting perioperative anemia, in order to minimize the transfusion of banked blood. Simultaneously, lung-protective ventilation strategies should be employed—including the monitoring of intra-abdominal pressure to prevent renal venous congestion—to reduce the incidence of postoperative AKI.

### 5.6 Elevated preoperative white blood cell count and lactate levels are risk factors for AKI

Studies have found that elevated preoperative white blood cell count and lactate levels are significantly associated with the risk of AKI. An elevated preoperative white blood cell count is a marker of activated systemic inflammation. In acute aortic dissection, the tear in the vascular wall itself can trigger the release of large amounts of inflammatory cytokines such as IL-6 and TNF-α (88), activating neutrophils and promoting their infiltration into renal tissue, where they release reactive oxygen species and proteases, directly damaging renal tubular epithelial cells (89, 90). Concurrently, the inflammatory response can induce endothelial dysfunction, increase vascular permeability, and lead to renal interstitial edema, which further compresses the renal tubules and microvessels, creating a vicious cycle (91, 92). Elevated preoperative lactate levels reflect inadequate tissue perfusion and increased anaerobic metabolism, indicating that the patient is in a pre-shock state or experiencing occult hypoperfusion. This microcirculatory dysfunction not only affects the kidneys but also foreshadows a reduced tolerance of systemic organs to subsequent stressors (93). All of this underscores the importance of preoperative monitoring and improvement of microcirculation.

## 6 Strengths and limitations

The strength of this study lies in its systematic and comprehensive approach. By searching eight major Chinese and English databases, the study included 44 studies involving a total of 11,983 patients and conducted an in-depth analysis of the relationship between 14 clinical variables—including age, BMI, hypertension, cardiopulmonary bypass time, and renal artery involvement, among 14 other clinical variables, with postoperative AKI following TAAD. A meta-analysis was conducted using a random-effects model, and publication bias was assessed through subgroup analysis, sensitivity analysis, and the trim-and-fill method, ensuring the robustness and clinical relevance of the results.

However, certain limitations remain. First, all included studies were retrospective in design, raising concerns about selection bias and residual confounding. Second, differences in AKI diagnostic criteria, perioperative management details, and variable definitions across studies may have led to high heterogeneity in results (e.g., *I*^2^ > 70% for CPB duration and BMI). Although sensitivity analyses were conducted, potential confounding factors could not be completely ruled out. Third, the Egger test indicated publication bias for six factors, including CPB duration and preoperative serum creatinine. Although the conclusions remained robust after correction using the trim-and-fill method, the effect sizes may have been overestimated. Fourth, meta-analysis could not be performed for some risk factors due to insufficient study numbers. Finally, as the majority of studies (90%) originated from China, caution is warranted when extrapolating the findings to other populations; meta-analysis could not be performed for some risk factors due to insufficient study numbers.

## 7 Conclusion

This meta-analysis revealed that the overall incidence of acute kidney injury following surgery for acute Stanford Type A aortic dissection was 50.72%, which was significantly higher than that observed in patients undergoing general cardiac surgery. Independent risk factors include: advancing age, male gender, high BMI, history of hypertension, elevated preoperative serum creatinine and cystatin C levels, elevated preoperative white blood cell count and lactate levels, renal artery involvement, prolonged cardiopulmonary bypass time, prolonged operative time, prolonged deep hypothermic circulatory arrest, increased perioperative red blood cell transfusion volume, and prolonged mechanical ventilation. Clinicians should optimize current preoperative assessment priorities and surgical perfusion strategies in response to these risk factors. Future multicenter prospective studies are needed to validate these findings. This will facilitate a shift in the management of postoperative AKI following TAAD from reactive treatment to proactive prevention.

## Data Availability

The data analyzed in this study is subject to the following licenses/restrictions: No restrictions apply. The data analyzed are derived from previously published studies cited in the reference list. No original datasets were generated. All extracted summary data (e.g., odds ratios, confidence intervals, incidence rates) are fully presented in the tables and figures of this article. Reasonable requests for further information can be directed to the corresponding author. Requests to access these datasets should be directed to Yu Yang, yangy6820@163.com.
