# Supplementary material for: Incidence and risk factors of acute kidney injury following Stanford type A aortic dissection surgery: a systematic review and meta-analysis
Source: Front Cardiovasc Med. 2026 Jun 15;13:1854958. doi: 10.3389/fcvm.2026.1854958 (PMC13310785; doi:10.3389/fcvm.2026.1854958)
Supplement: Supplementary file 1 [file Datasheet1.docx]

Supplementary materials

Table S1 Literature search strategy

1. Pubmed

| **Search number** | **Query** | **Sort By** | **Filters** | **Search Details** | **Results** | **Time** |
| --- | --- | --- | --- | --- | --- | --- |
| **1** | "Aortic Dissection"[Mesh] | Most Recent |  | "Aortic Dissection"[MeSH Terms] | 23,161 | 04:48:43 |
| **2** | "Acute Kidney Injury"[Mesh] | Most Recent |  | "Acute Kidney Injury"[MeSH Terms] | 62,674 | 04:51:00 |
| **3** | Aortic Dissections[Title/Abstract] OR Dissecting Aneurysms[Title/Abstract] OR Dissecting Aneurysm[Title/Abstract] OR Dissecting Aneurysm Aorta[Title/Abstract] OR Dissecting Aneurysm Aortas[Title/Abstract] OR Aortic Dissecting Aneurysm[Title/Abstract] OR Aortic Dissecting Aneurysms[Title/Abstract] | Most Recent |  | "aortic dissections"[Title/Abstract] OR "dissecting aneurysms"[Title/Abstract] OR "dissecting aneurysm"[Title/Abstract] OR "dissecting aneurysm aorta"[Title/Abstract] OR (("dissect"[All Fields] OR "dissected"[All Fields] OR "Dissecting"[All Fields] OR "dissection"[MeSH Terms] OR "dissection"[All Fields] OR "Dissections"[All Fields] OR "dissects"[All Fields]) AND "aneurysm aortas"[Title/Abstract]) OR "aortic dissecting aneurysm"[Title/Abstract] OR "aortic dissecting aneurysms"[Title/Abstract] | 5,718 | 04:51:47 |
| **4** | Acute Kidney Injuries[Title/Abstract] OR Acute Renal Injury[Title/Abstract] OR Acute Renal Injuries[Title/Abstract] OR Acute Kidney Failures[Title/Abstract] OR Acute Kidney Failure[Title/Abstract] OR Acute Renal Failure[Title/Abstract] OR Acute Renal Failures[Title/Abstract] OR Acute Renal Insufficiencies[Title/Abstract] OR Acute Kidney Insufficiency[Title/Abstract] OR Acute Renal Insufficiency[Title/Abstract] OR Acute Kidney Insufficiencies[Title/Abstract] | Most Recent |  | "acute kidney injuries"[Title/Abstract] OR "acute renal injury"[Title/Abstract] OR "acute renal injuries"[Title/Abstract] OR "acute kidney failures"[Title/Abstract] OR "acute kidney failure"[Title/Abstract] OR "acute renal failure"[Title/Abstract] OR "acute renal failures"[Title/Abstract] OR "acute renal insufficiencies"[Title/Abstract] OR "acute kidney insufficiency"[Title/Abstract] OR "acute renal insufficiency"[Title/Abstract] OR "acute kidney insufficiencies"[Title/Abstract] | 30,853 | 04:52:09 |
| **5** | ("Aortic Dissection"[Mesh]) OR (Aortic Dissections[Title/Abstract] OR Dissecting Aneurysms[Title/Abstract] OR Dissecting Aneurysm[Title/Abstract] OR Dissecting Aneurysm Aorta[Title/Abstract] OR Dissecting Aneurysm Aortas[Title/Abstract] OR Aortic Dissecting Aneurysm[Title/Abstract] OR Aortic Dissecting Aneurysms[Title/Abstract]) | Most Recent |  | "Aortic Dissection"[MeSH Terms] OR ("aortic dissections"[Title/Abstract] OR "dissecting aneurysms"[Title/Abstract] OR "dissecting aneurysm"[Title/Abstract] OR "dissecting aneurysm aorta"[Title/Abstract] OR (("dissect"[All Fields] OR "dissected"[All Fields] OR "Dissecting"[All Fields] OR "dissection"[MeSH Terms] OR "dissection"[All Fields] OR "Dissections"[All Fields] OR "dissects"[All Fields]) AND "aneurysm aortas"[Title/Abstract]) OR "aortic dissecting aneurysm"[Title/Abstract] OR "aortic dissecting aneurysms"[Title/Abstract]) | 25,352 | 04:52:26 |
| **6** | ("Acute Kidney Injury"[Mesh]) OR (Acute Kidney Injuries[Title/Abstract] OR Acute Renal Injury[Title/Abstract] OR Acute Renal Injuries[Title/Abstract] OR Acute Kidney Failures[Title/Abstract] OR Acute Kidney Failure[Title/Abstract] OR Acute Renal Failure[Title/Abstract] OR Acute Renal Failures[Title/Abstract] OR Acute Renal Insufficiencies[Title/Abstract] OR Acute Kidney Insufficiency[Title/Abstract] OR Acute Renal Insufficiency[Title/Abstract] OR Acute Kidney Insufficiencies[Title/Abstract]) | Most Recent |  | "Acute Kidney Injury"[MeSH Terms] OR "acute kidney injuries"[Title/Abstract] OR "acute renal injury"[Title/Abstract] OR "acute renal injuries"[Title/Abstract] OR "acute kidney failures"[Title/Abstract] OR "acute kidney failure"[Title/Abstract] OR "acute renal failure"[Title/Abstract] OR "acute renal failures"[Title/Abstract] OR "acute renal insufficiencies"[Title/Abstract] OR "acute kidney insufficiency"[Title/Abstract] OR "acute renal insufficiency"[Title/Abstract] OR "acute kidney insufficiencies"[Title/Abstract] | 75,699 | 04:52:44 |
| **7** | #5 AND #6 |  |  | ("Aortic Dissection"[MeSH Terms] OR ("aortic dissections"[Title/Abstract] OR "dissecting aneurysms"[Title/Abstract] OR "dissecting aneurysm"[Title/Abstract] OR "dissecting aneurysm aorta"[Title/Abstract] OR (("dissect"[All Fields] OR "dissected"[All Fields] OR "Dissecting"[All Fields] OR "dissection"[MeSH Terms] OR "dissection"[All Fields] OR "Dissections"[All Fields] OR "dissects"[All Fields]) AND "aneurysm aortas"[Title/Abstract]) OR "aortic dissecting aneurysm"[Title/Abstract] OR "aortic dissecting aneurysms"[Title/Abstract])) AND ("Acute Kidney Injury"[MeSH Terms] OR ("acute kidney injuries"[Title/Abstract] OR "acute renal injury"[Title/Abstract] OR "acute renal injuries"[Title/Abstract] OR "acute kidney failures"[Title/Abstract] OR "acute kidney failure"[Title/Abstract] OR "acute renal failure"[Title/Abstract] OR "acute renal failures"[Title/Abstract] OR "acute renal insufficiencies"[Title/Abstract] OR "acute kidney insufficiency"[Title/Abstract] OR "acute renal insufficiency"[Title/Abstract] OR "acute kidney insufficiencies"[Title/Abstract])) | 317 | 04:56:40 |

1. Embase

| No. | Query | Results | Date |
| --- | --- | --- | --- |
| #1. | 'aortic dissection'/exp | 34,991 | 6-Jan-26 |
| #2. | aortic dissections':ti,ab,kw OR 'dissecting aneurysms':ti,ab,kw OR 'dissecting aneurysm':ti,ab,kw OR 'dissecting aneurysm aorta':ti,ab,kw OR 'dissecting aneurysm aortas':ti,ab,kw OR 'aortic dissecting aneurysm':ti,ab,kw OR 'aortic dissecting aneurysms':ti,ab,kw | 7,530 | 6-Jan-26 |
| #3. | 'acute kidney failure'/exp | 160,797 | 6-Jan-26 |
| #4. | acute kidney injuries':ti,ab,kw OR 'acute renal injury':ti,ab,kw OR 'acute renal failures':ti,ab,kw OR 'acute kidney injuries':ti,ab,kw OR 'acute kidney failure':ti,ab,kw OR 'acute renal failure':ti,ab,kw OR 'acute renal failures':ti,ab,kw OR 'acute renal insufficiencies':ti,ab,kw OR 'acute kidney insufficiency':ti,ab,kw OR 'acute renal insufficiency':ti,ab,kw OR 'acute kidney insufficiencies':ti,ab,kw | 160,797 | 6-Jan-26 |
| #5. | #1 OR #2 | 39,215 | 6-Jan-26 |
| #6. | #3 OR #4 | 171,751 | 6-Jan-26 |
| #7. | #5 AND #6 | 1,490 | 6-Jan-26 |

1. Cochrane

| ID Search Hits |
| --- |
| #1 MeSH descriptor: [Aortic Dissection] explode all trees 268 |
| #2 MeSH descriptor: [Acute Kidney Injury] explode all trees 2571 |
| #3 (Aortic Dissections):ti,ab,kw OR (Dissecting Aneurysms):ti,ab,kw OR (Dissecting Aneurysm):ti,ab,kw OR (Dissecting Aneurysm Aorta):ti,ab,kw OR (Dissecting Aneurysm Aortas):ti,ab,kw OR (Aortic Dissecting Aneurysm):ti,ab,kw OR (Aortic Dissecting Aneurysms):ti,ab,kw 142 |
| #4 (Acute Kidney Injuries):ti,ab,kw OR (Acute Renal Injury):ti,ab,kw OR (Acute Renal Injuries):ti,ab,kw OR (Acute Kidney Failures):ti,ab,kw OR (Acute Kidney Failure):ti,ab,kw OR (Acute Renal Failure):ti,ab,kw OR (Acute Renal Failures):ti,ab,kw OR (Acute Renal Insufficiencies):ti,ab,kw OR (Acute Kidney Insufficiency):ti,ab,kw OR (Acute Renal Insufficiency):ti,ab,kw OR (Acute Kidney Insufficiencies):ti,ab,kw 12291 |
| #5 #1 OR #3 362 |
| #6 #2 OR #4 12681 |
| #7 #5 AND #6 30 |

1. Web of science

| No. | Searches | Results | Date Run |
| --- | --- | --- | --- |
| #1 | TS=(Aortic Dissections OR Dissecting Aneurysms OR Dissecting Aneurysm OR Dissecting Aneurysm Aorta OR Dissecting Aneurysm Aortas OR Aortic Dissecting Aneurysm OR Aortic Dissecting Aneurysms) | 20299 | Mon Jan 05 2026 18:25:15 GMT+0800 (中国标准时间) |
| #2 | TS=(Acute Kidney Injuries OR Acute Renal Injury OR Acute Renal Injuries OR Acute Kidney Failures OR Acute Kidney Failure OR Acute Renal Failure OR Acute Renal Failures OR Acute Renal Insufficiencies OR Acute Kidney Insufficiency OR Acute Renal Insufficiency OR Acute Kidney Insufficiencies) | 79339 | Mon Jan 05 2026 18:25:54 GMT+0800 (中国标准时间) |
| #3 | #1 AND #2 | 822 | Mon Jan 05 2026 18:26:16 GMT+0800 (中国标准时间) |

1. China Knowledge Resource Integrated Database (CNKI)

| SU=(A型主动脉夹层 + 主动脉夹层 + 主动脉瘤 + 夹层动脉瘤) AND SU=(急性肾功能衰竭 + 急性肾脏功能衰竭 + 急性肾损伤 + 急性肾脏功能不全 + 急性肾功能不全) |
| --- |
| 197 |

1. Wangfang Database

| 题名或关键词: (A型主动脉夹层 or 主动脉夹层 or 主动脉瘤 or 夹层动脉瘤) and 题名或关键词: (急性肾功能衰竭 or 急性肾脏功能衰竭 or 急性肾损伤 or 急性肾脏功能不全 or 急性肾功能不全) |
| --- |
| 438 |

1. Weipu Database (VIP)

| M=(Ａ型主动脉夹层 OR 主动脉夹层 OR 主动脉瘤 OR 夹层动脉瘤) AND M=(急性肾功能衰竭 OR 急性肾脏功能衰竭 OR 急性肾损伤 OR 急性肾脏功能不全 OR 急性肾功能不全) |
| --- |
| 154 |

1. Chinese Biomedical Database (CBM)

| ("急性肾功能衰竭"[常用字段:智能] OR "急性肾脏功能衰竭"[常用字段:智能] OR "急性肾损伤"[常用字段:智能] OR "急性肾脏功能不全"[常用字段:智能] OR "急性肾功能不全"[常用字段:智能]) AND ("A型主动脉夹层"[常用字段:智能] OR "主动脉夹层"[常用字段:智能] OR "主动脉瘤"[常用字段:智能] OR "夹层动脉瘤"[常用字段:智能]) |
| --- |
| 409 |


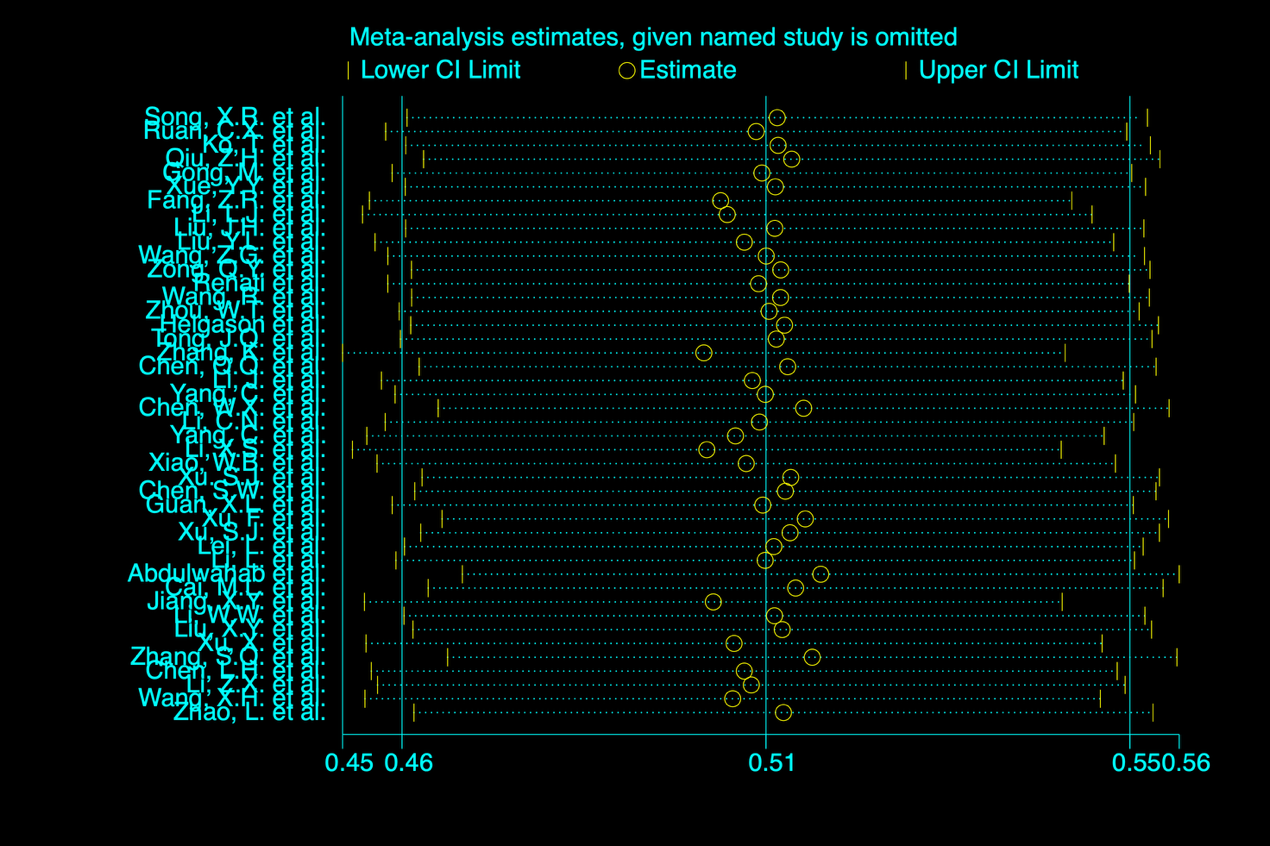


Figure S1 Sensitivity analysis plot of the incidence of AKI


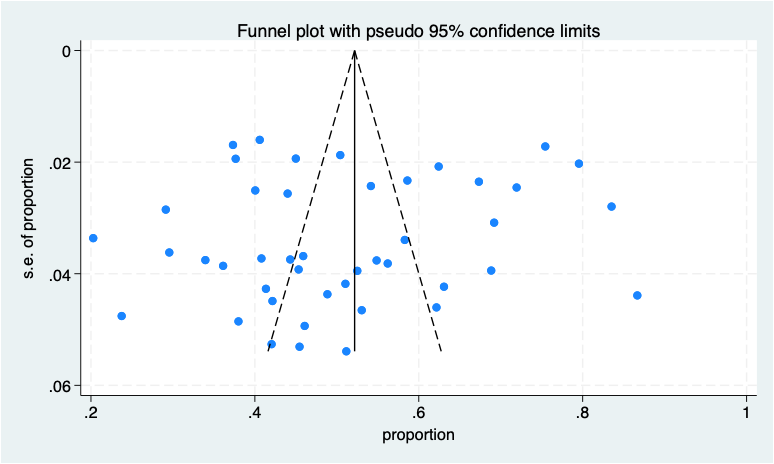


Figure S2 Funnel plot of meta-analysis of the incidence of AKI.


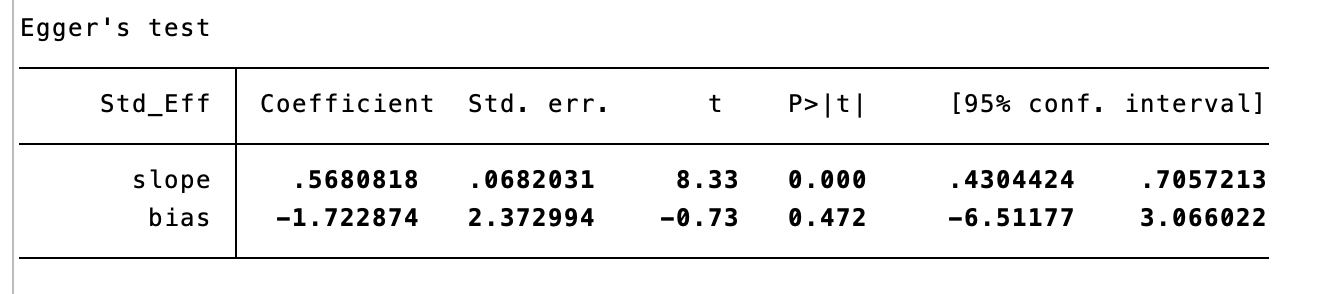


Figure S3 Results of egger’s test of the incidence of AKI.


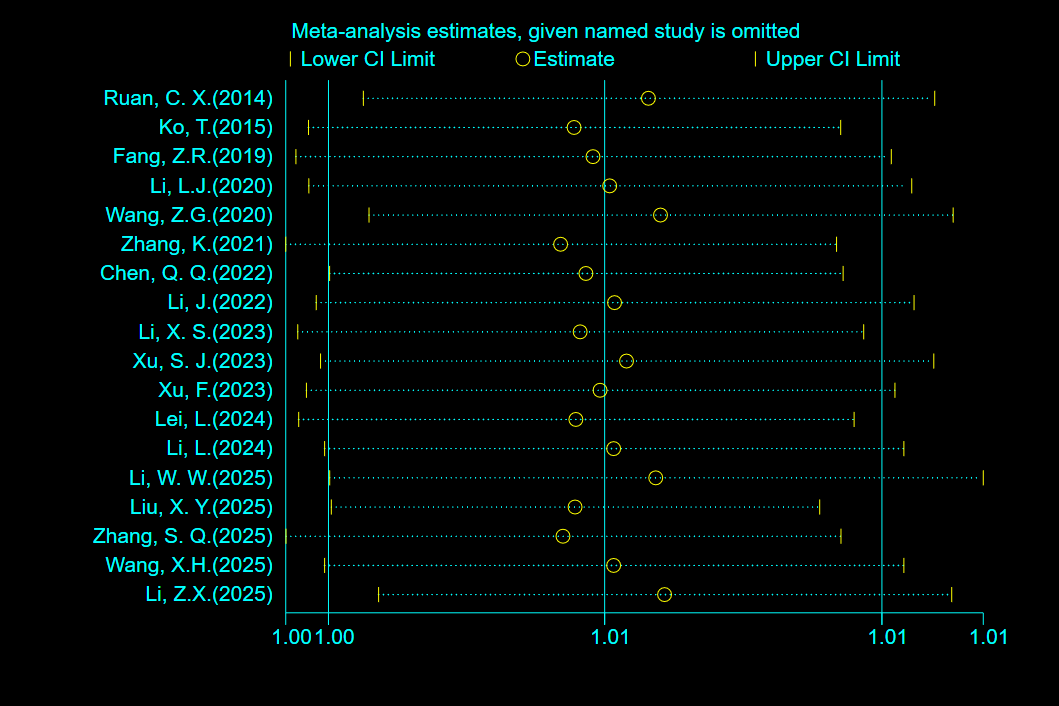


Figure S4 Sensitivity analysis plot of prolonged cardiopulmonary bypass.


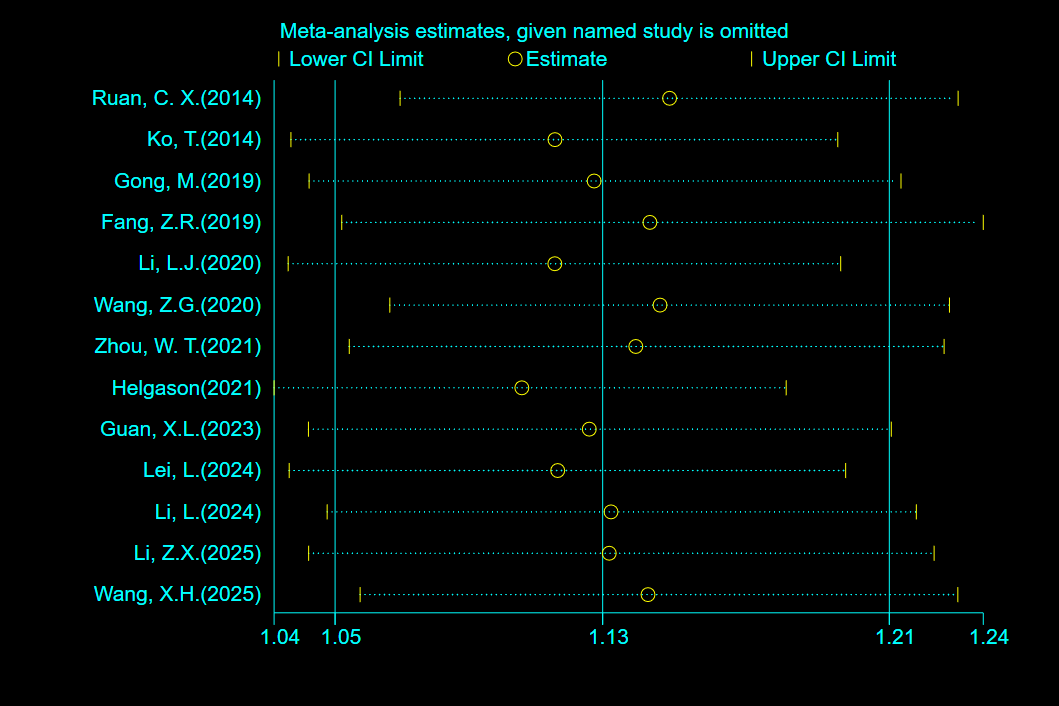


Figure S5 Sensitivity analysis plot of BMI.


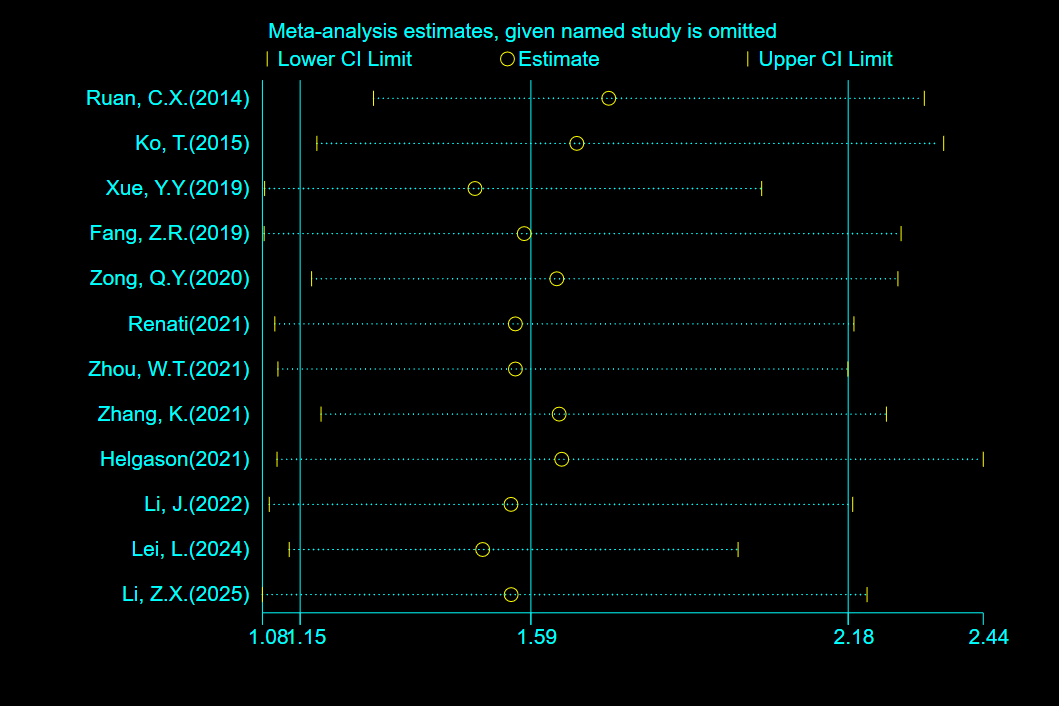


Figure S6 Sensitivity analysis plot of history of hypertension.


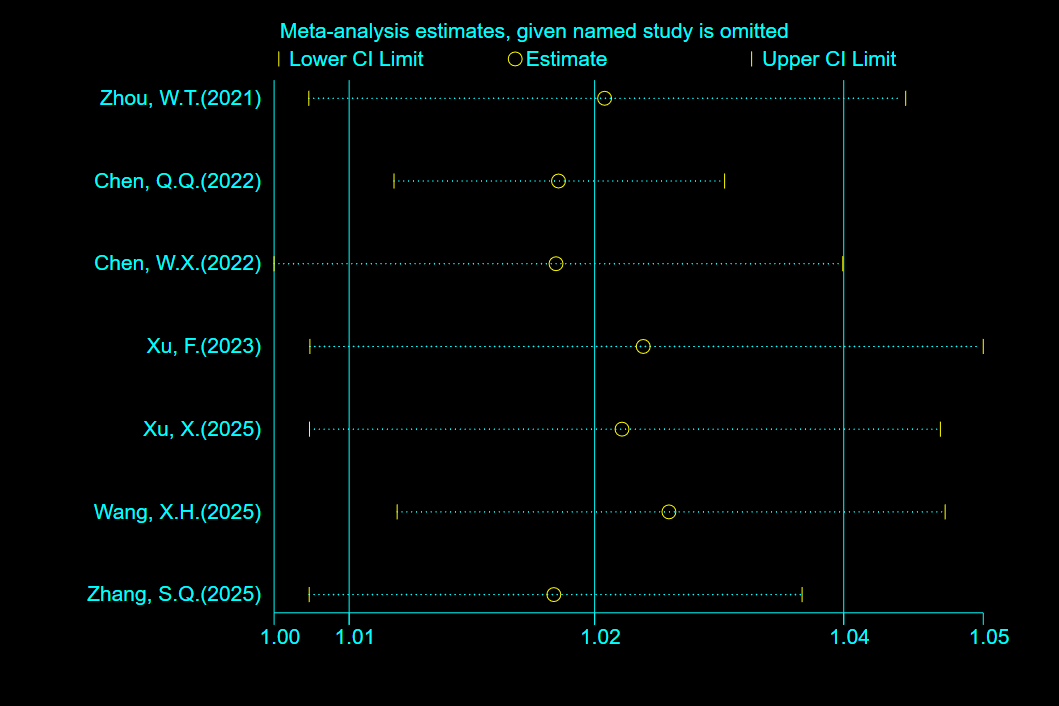


Figure S7 Sensitivity analysis plot of preoperative serum creatinine


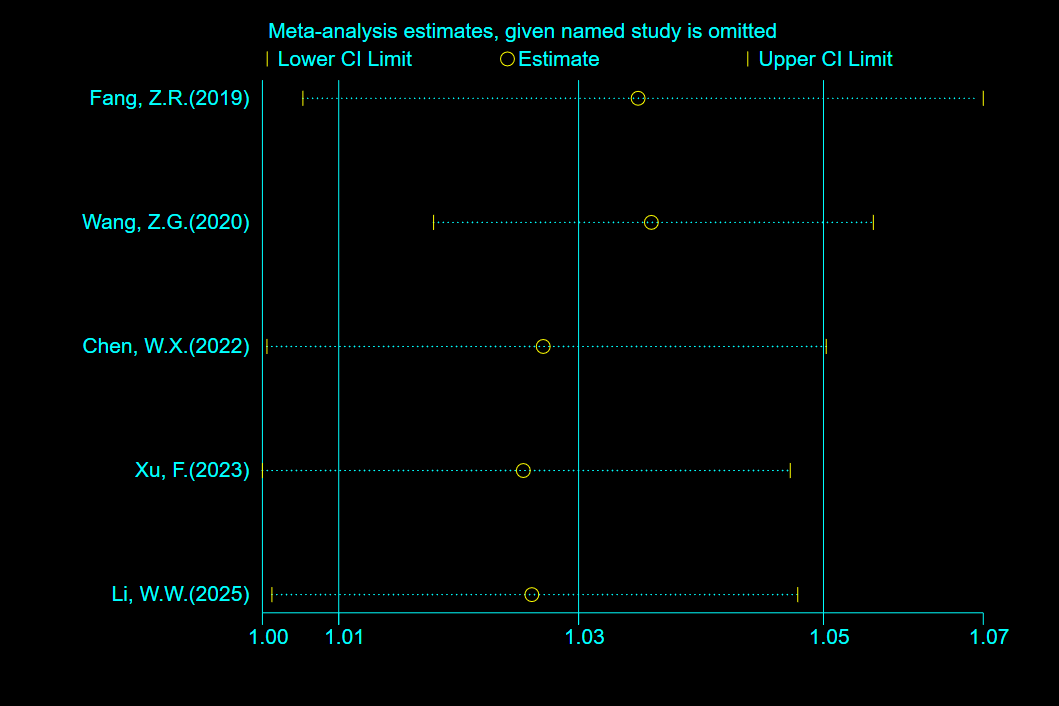


Figure S8 Sensitivity analysis plot of age (per 1 year)


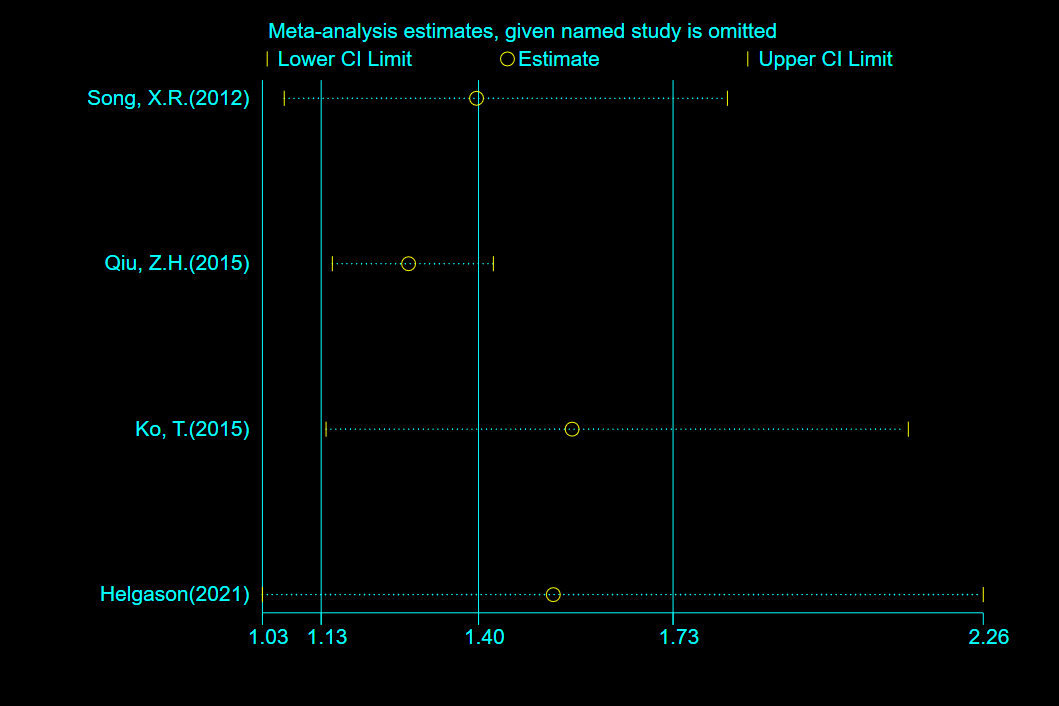


Figure S9 Sensitivity analysis plot of age (per 10 years)


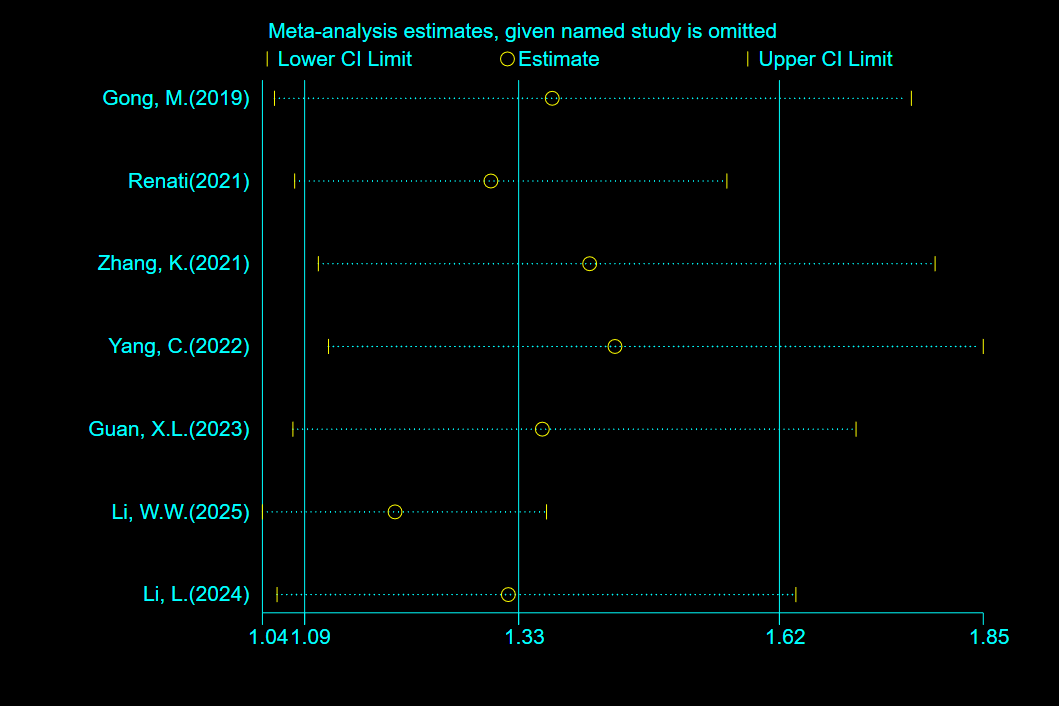


Figure S10 Sensitivity analysis plot of prolonged duration of surgery.


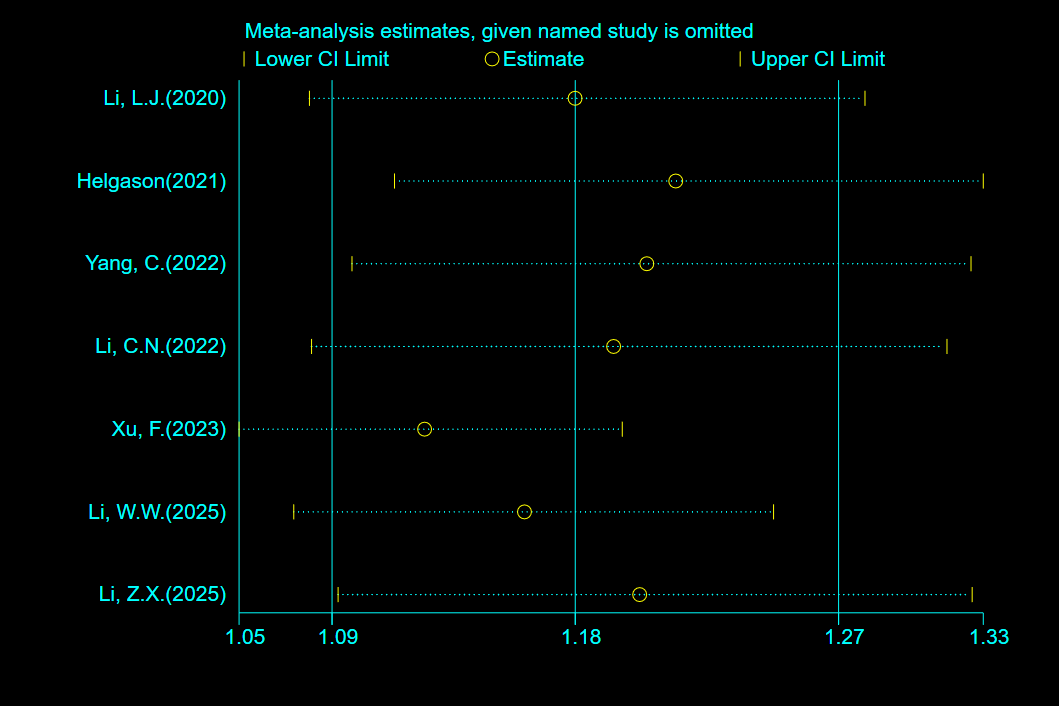


Figure S11 Sensitivity analysis plot of the volume of perioperative red blood cell transfusion.


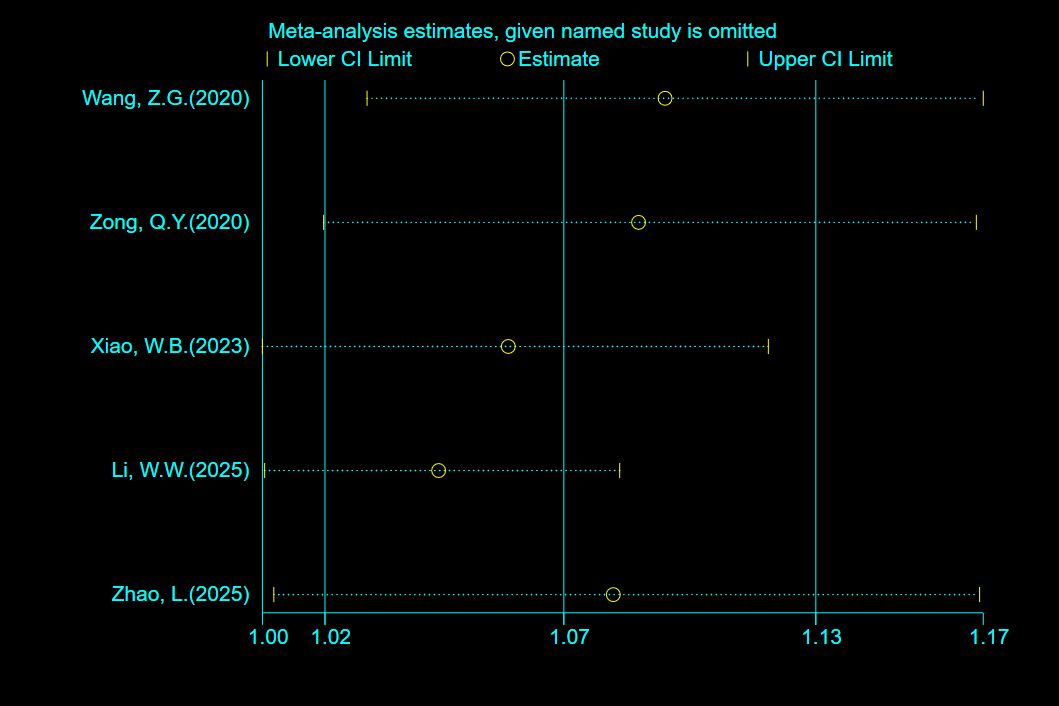


Figure S12 Sensitivity analysis plot of the duration of deep hypothermic circulatory arrest.


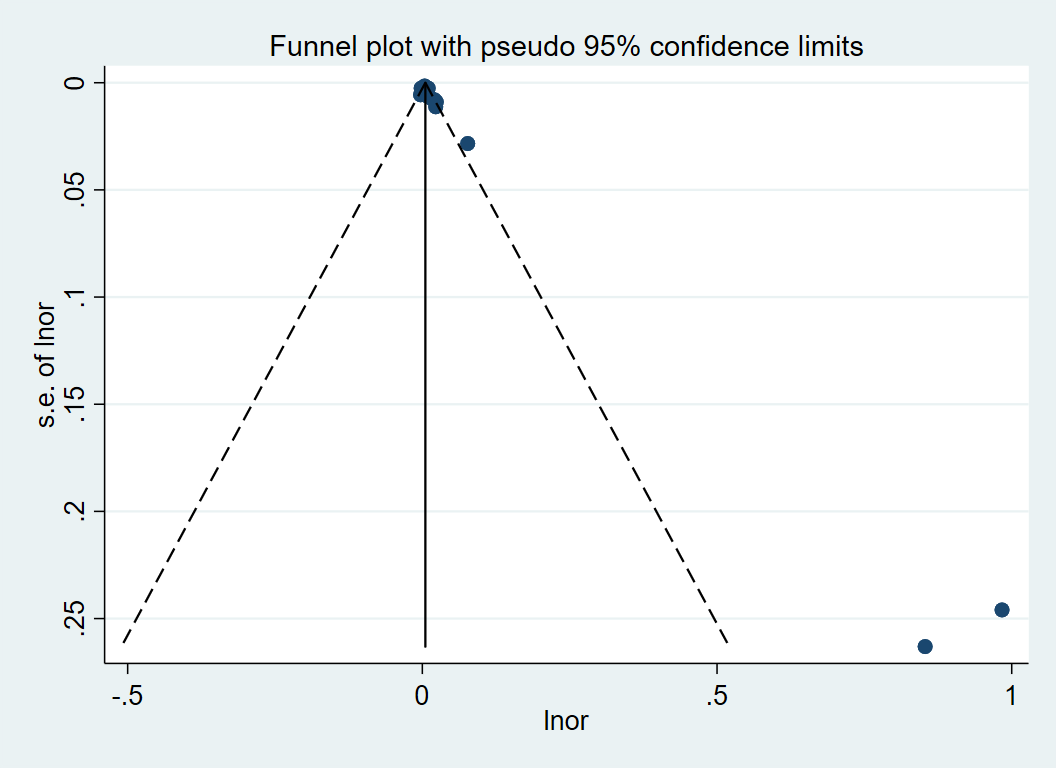


Figure S13 Funnel plot of meta-analysis of prolonged cardiopulmonary bypass.


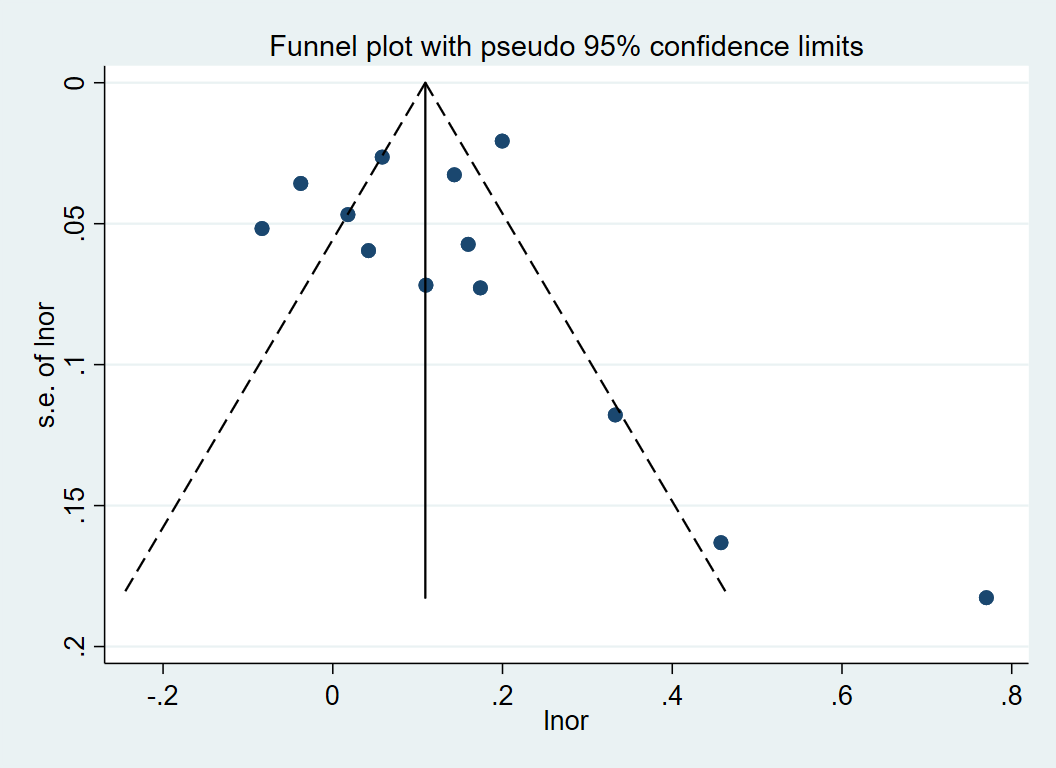


Figure S14 Funnel plot of meta-analysis of BMI.


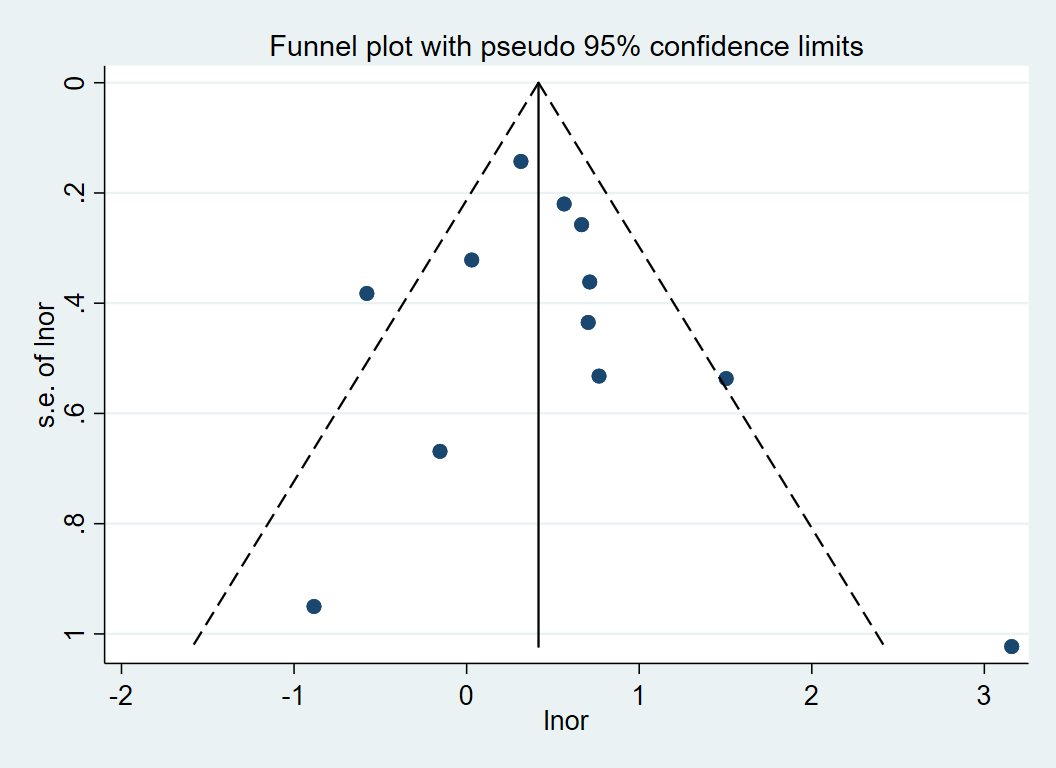


Figure S15 Funnel plot of meta-analysis of history of hypertension.


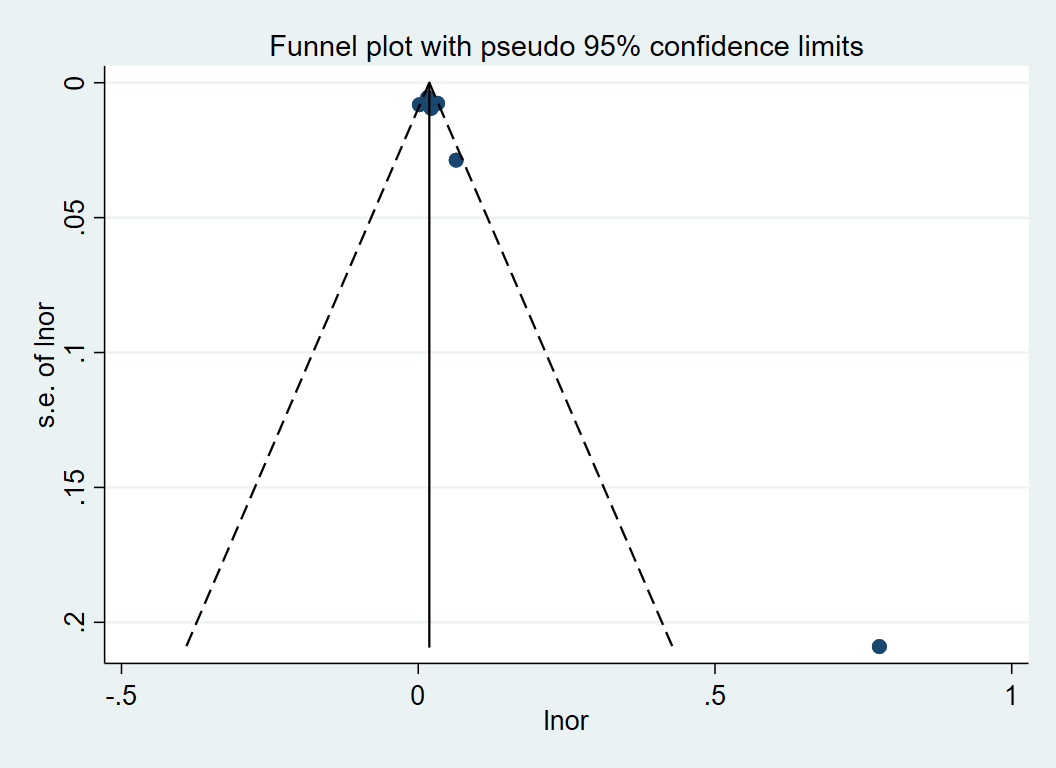


Figure S16 Funnel plot of meta-analysis of preoperative serum creatinine.


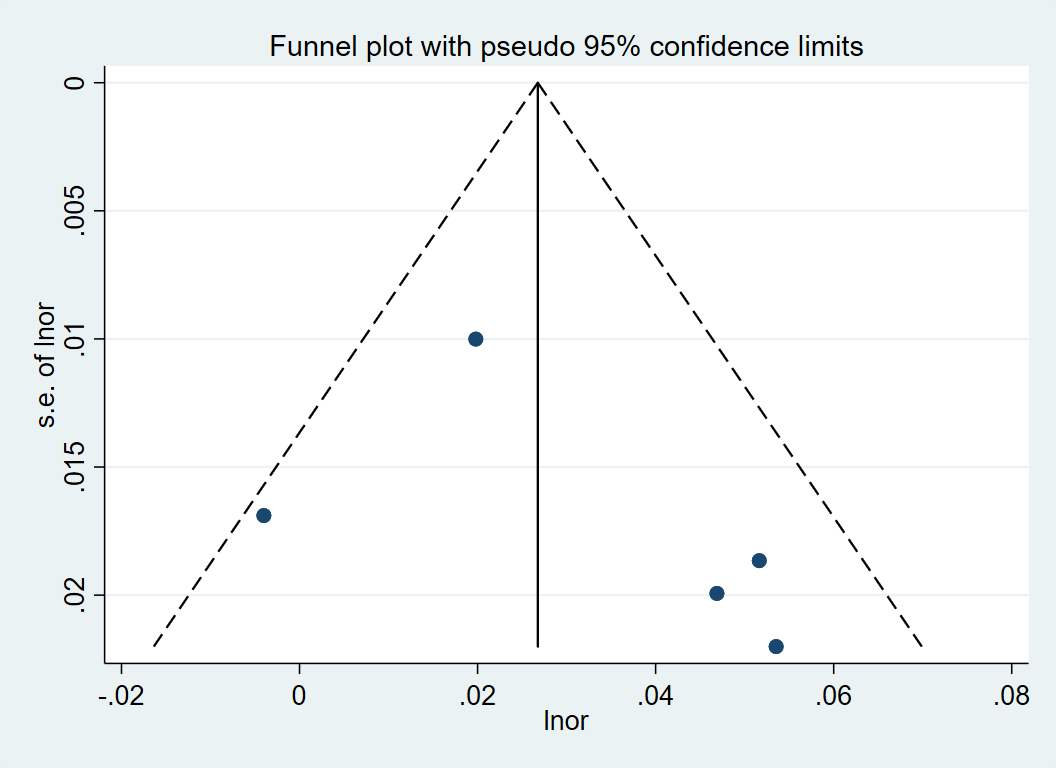


Figure S17 Funnel plot of meta-analysis of age (per 1 year).


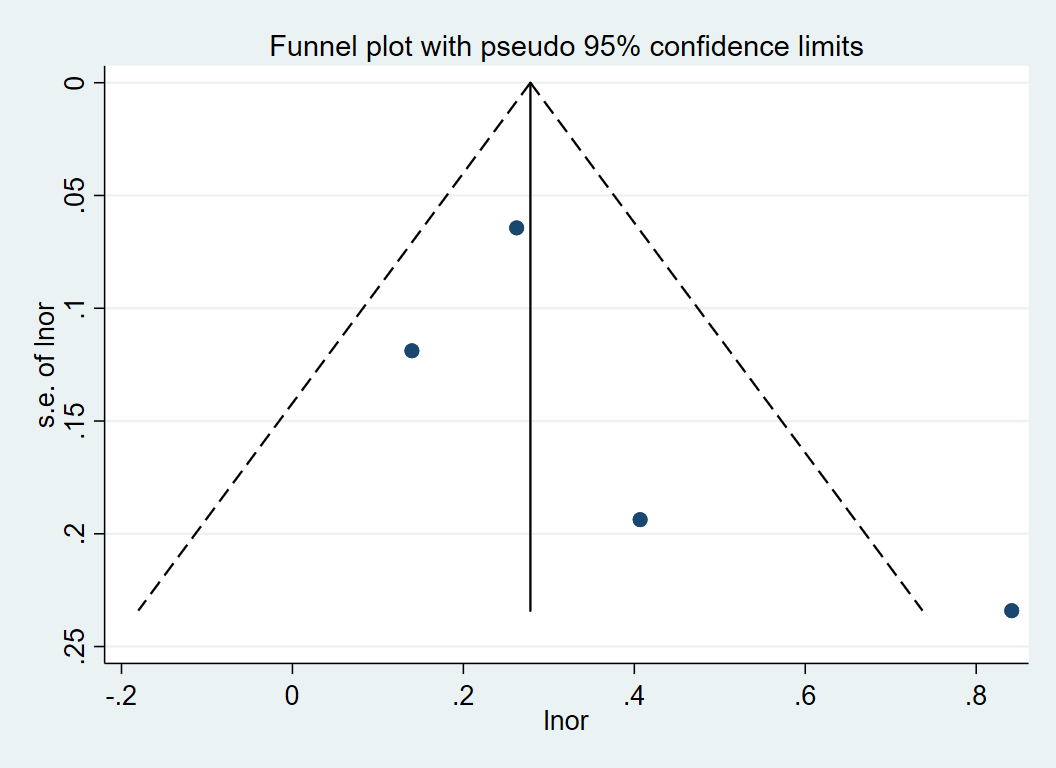


Figure S18 Funnel plot of meta-analysis of age (per 10 year).


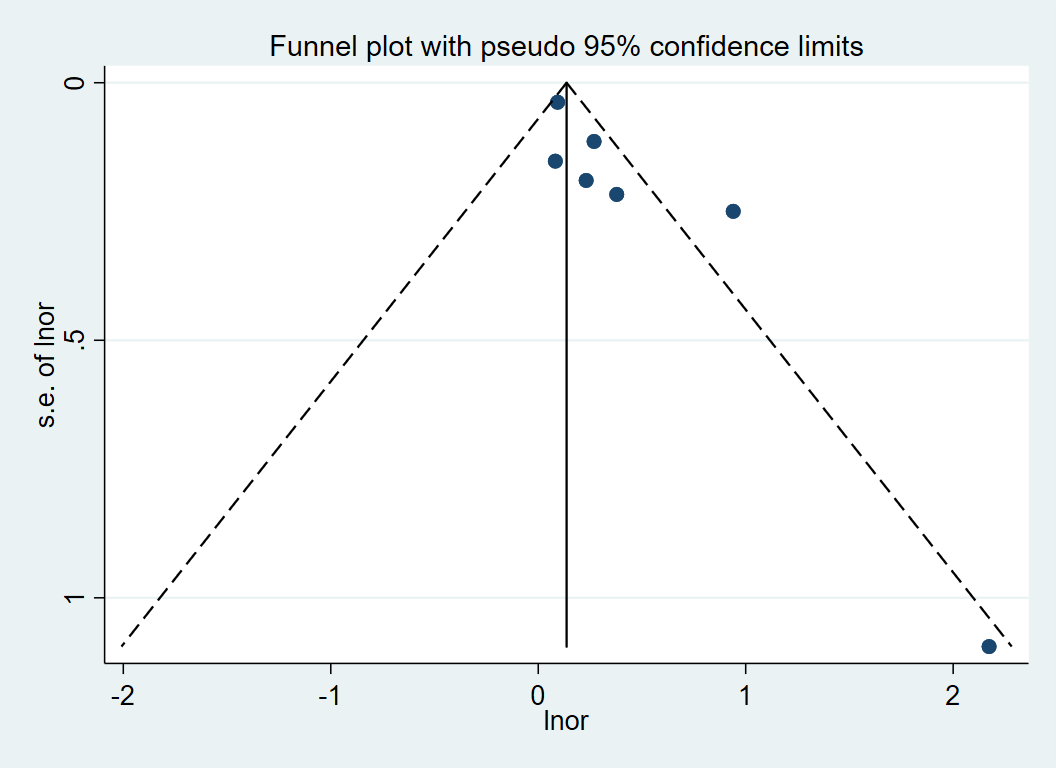


Figure S19 Funnel plot of meta-analysis of prolonged duration of surgery.


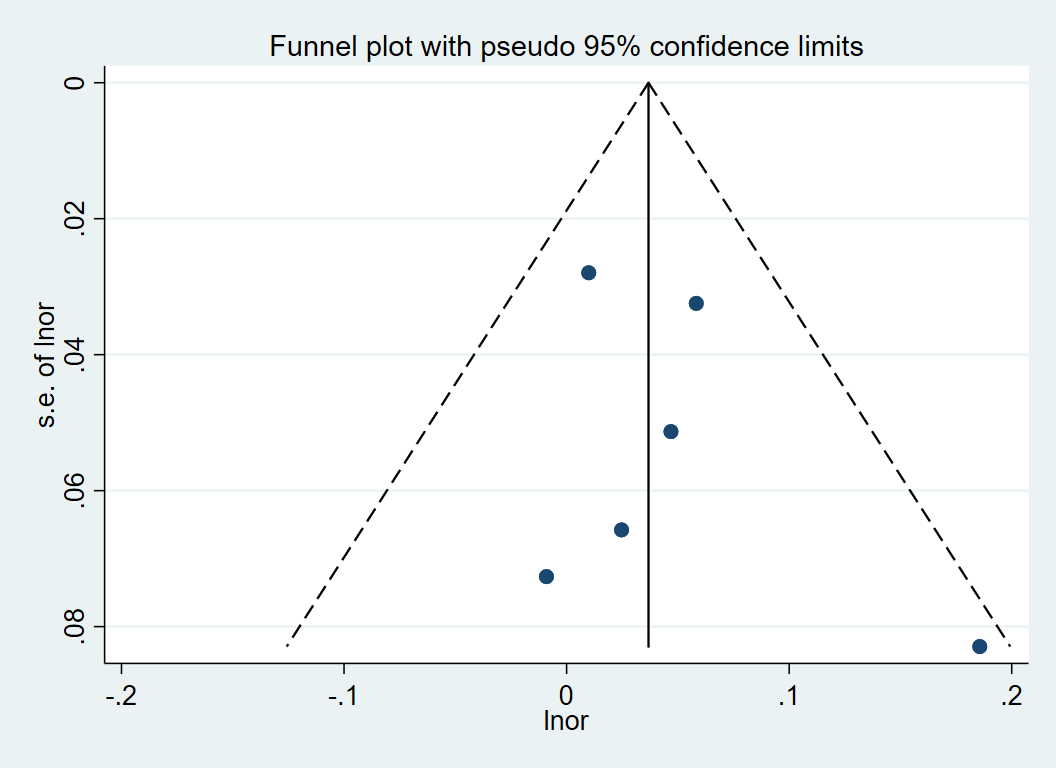


Figure S20 Funnel plot of meta-analysis of preoperative leukocyte counts.


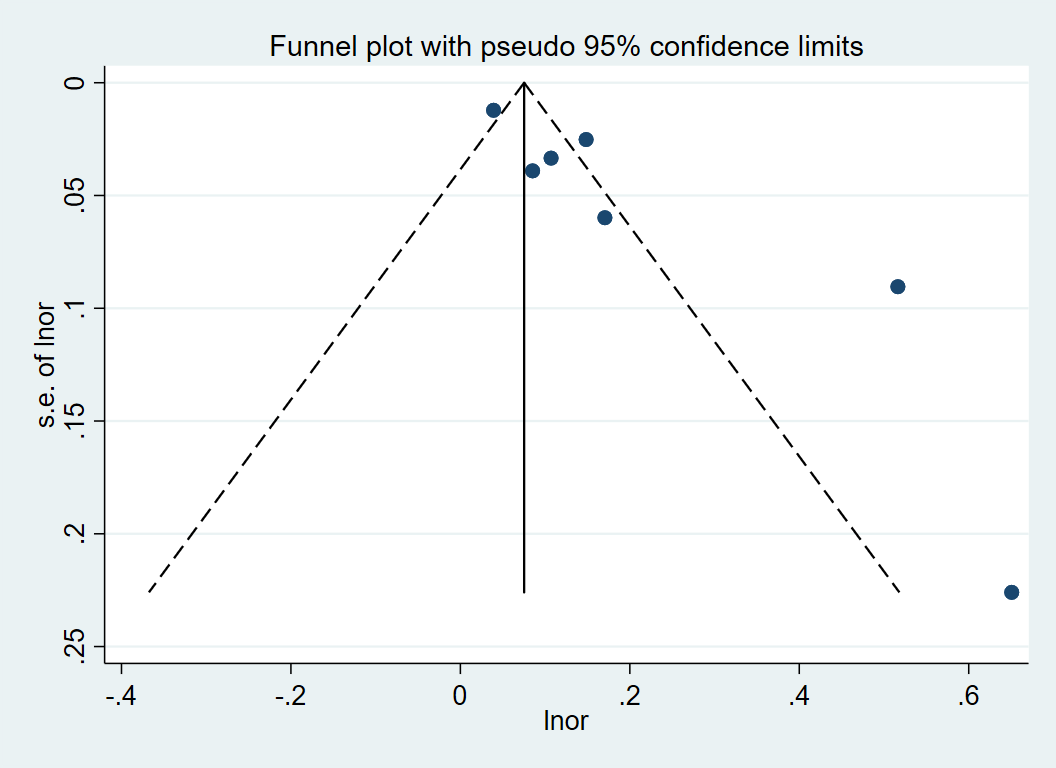


Figure S21 Funnel plot of meta-analysis of the volume of perioperative red blood cell transfusion.


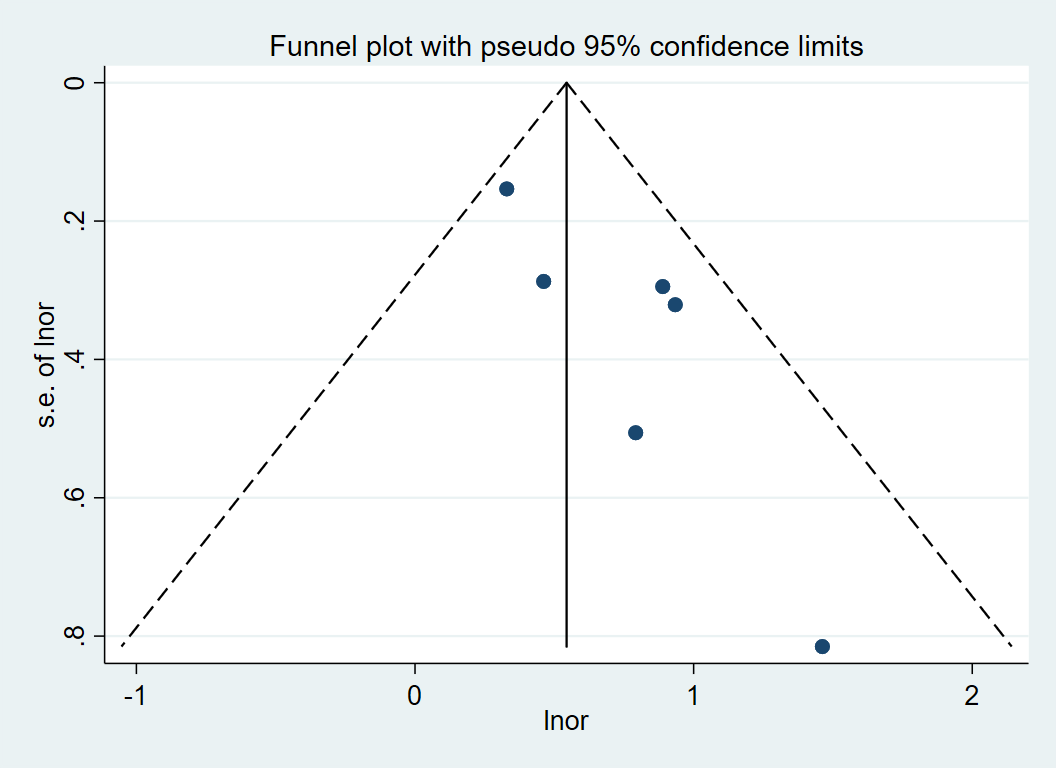


Figure S22 Funnel plot of meta-analysis of male.


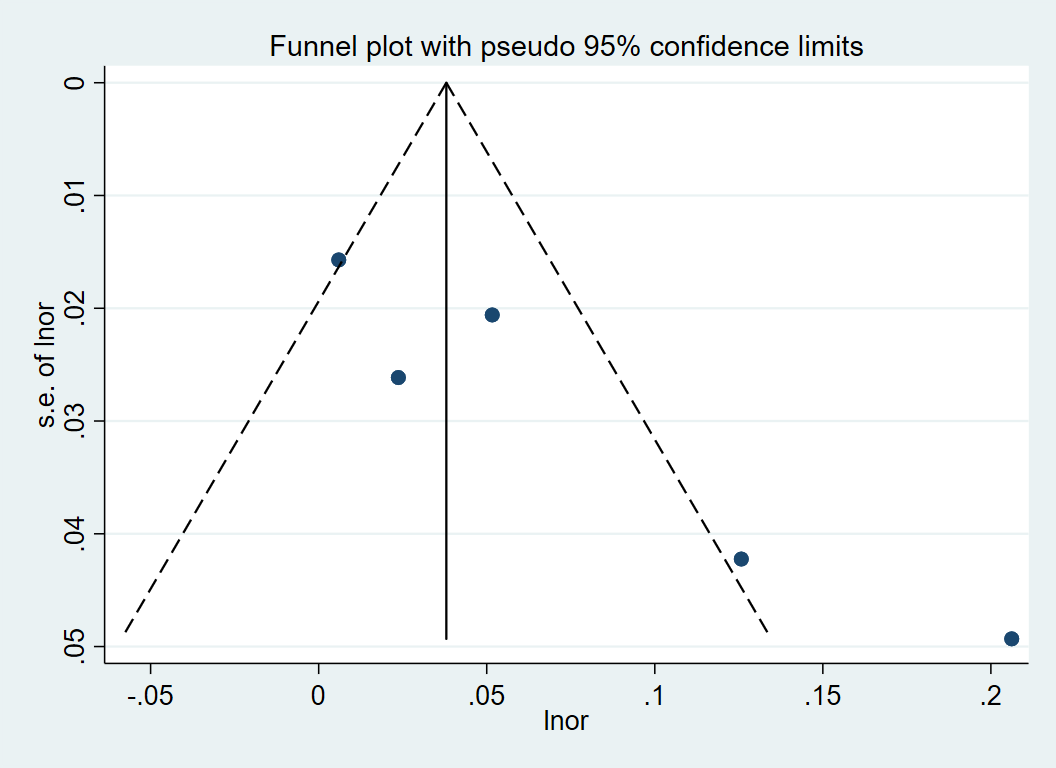


Figure S23 Funnel plot of meta-analysis of the duration of deep hypothermic circulatory arrest.


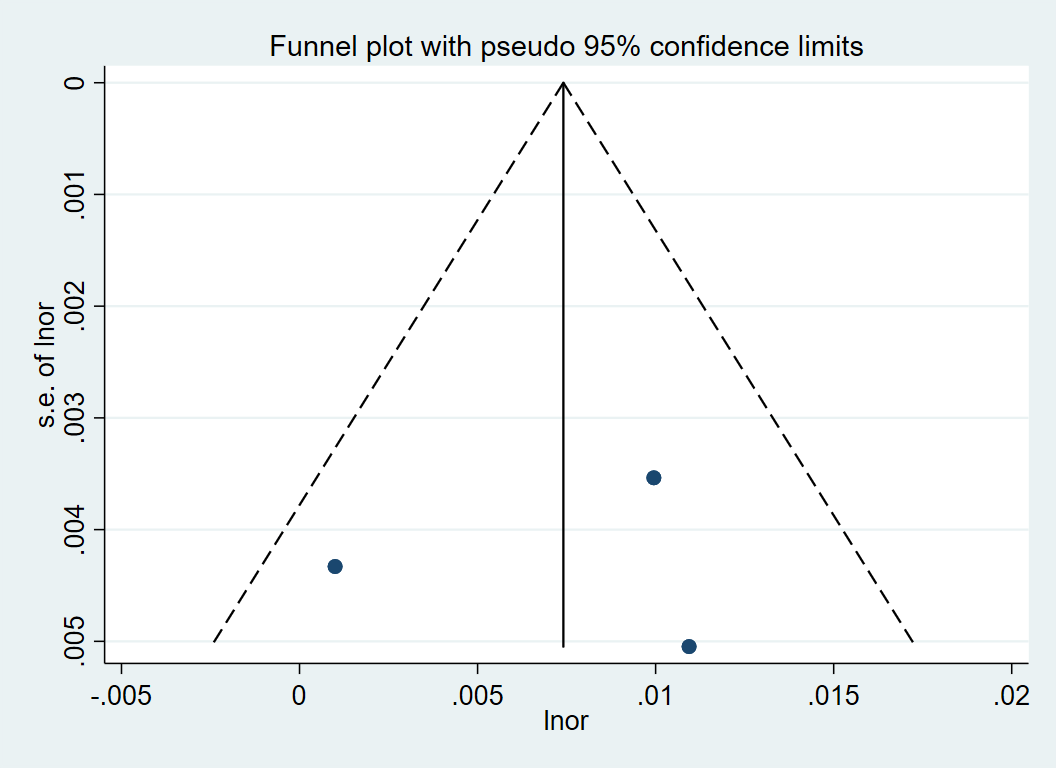


Figure S24 Funnel plot of meta-analysis of the duration of perioperative mechanical ventilation.


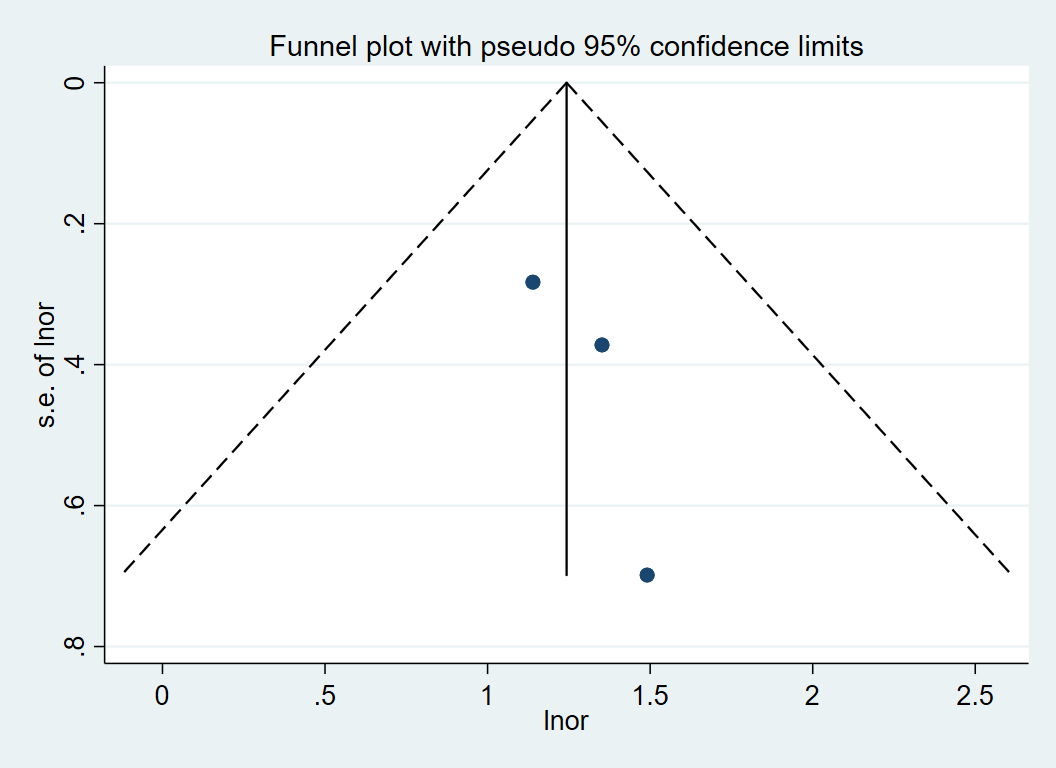


Figure S25 Funnel plot of meta-analysis of preoperative dissection involving the renal artery.


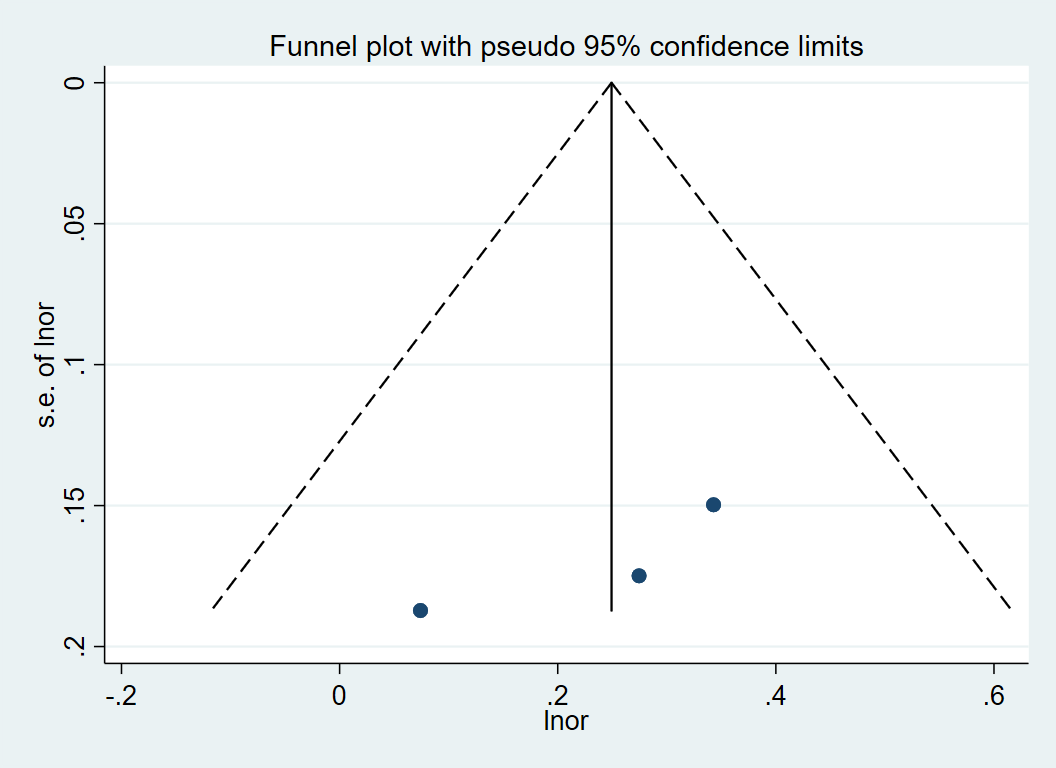


Figure S26 Funnel plot of meta-analysis of preoperative lactate levels.


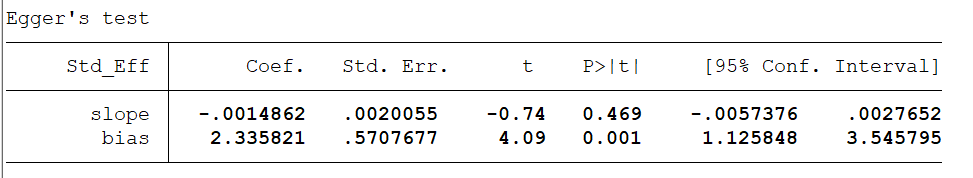


Figure S27 Results of egger’s test of prolonged cardiopulmonary bypass.


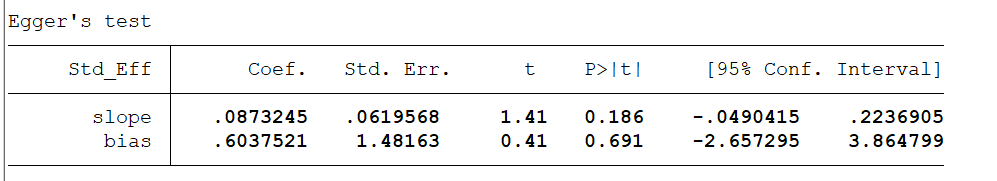


Figure S28 Results of egger’s test of BMI.


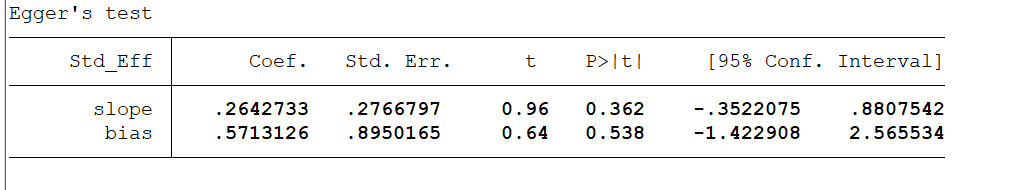


Figure S29 Results of egger’s test of history of hypertension.


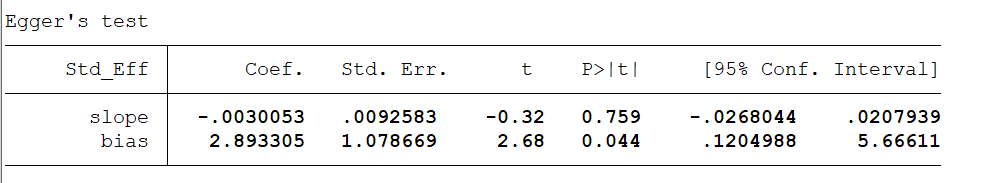


Figure S30 Results of egger’s test of preoperative serum creatinine.


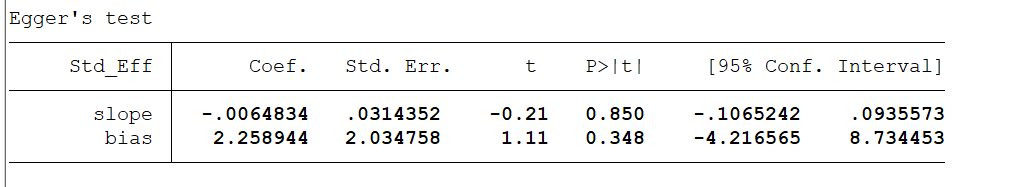


Figure S31 Results of egger’s test of age (per 1 year).


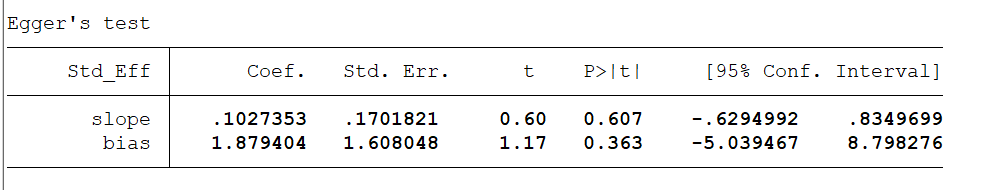


Figure S32 Results of egger’s test of age (per 10 years).


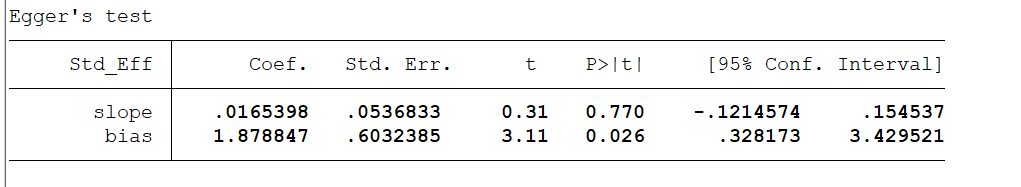


Figure S33 Results of egger’s test of prolonged duration of surgery.


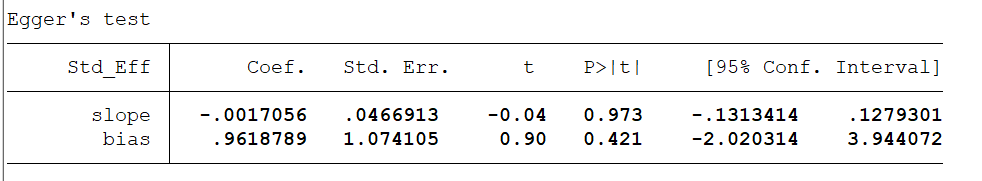


Figure S34 Results of egger’s test of preoperative leukocyte counts.


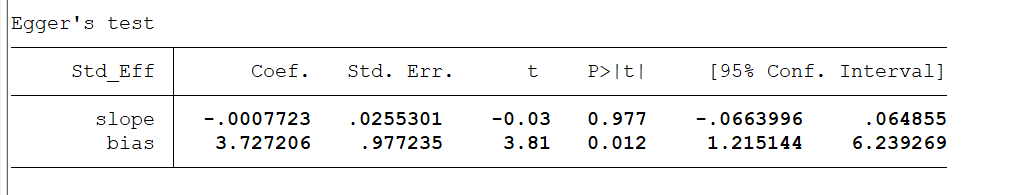
 Figure S35 Results of egger’s test of the volume of perioperative red blood cell transfusion.


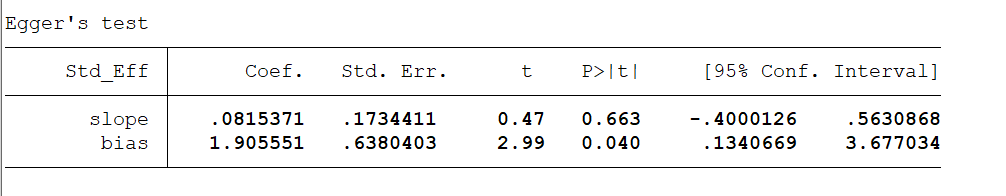


Figure S36 Results of egger’s test of the volume of male.


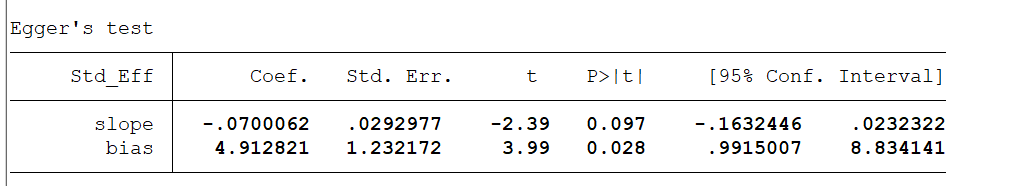
 Figure S37 Results of egger’s test of the duration of deep hypothermic circulatory arrest.


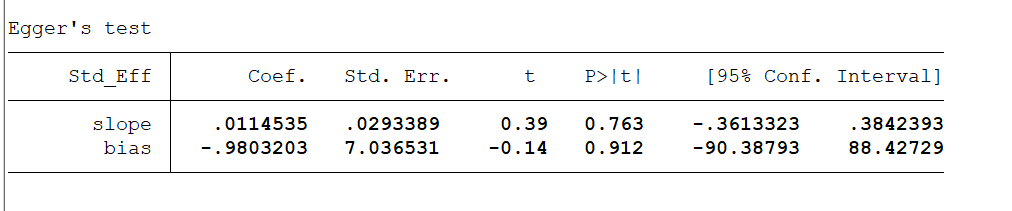
 Figure S38 Results of egger’s test of the duration of perioperative mechanical ventilation.


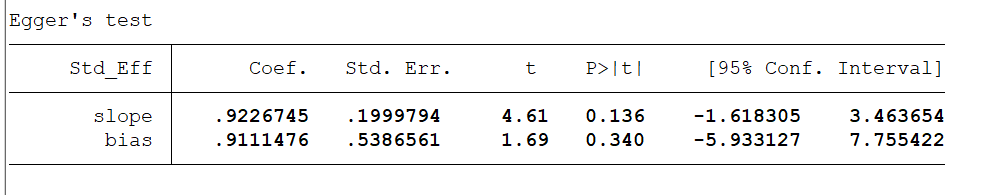


Figure S39 Results of egger’s test of preoperative dissection involving the renal artery.


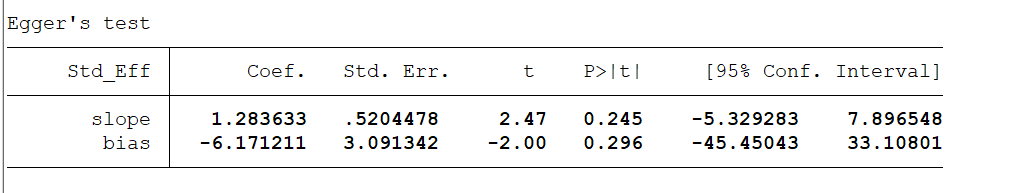
 Figure S40 Results of egger’s test of the preoperative lactate levels.


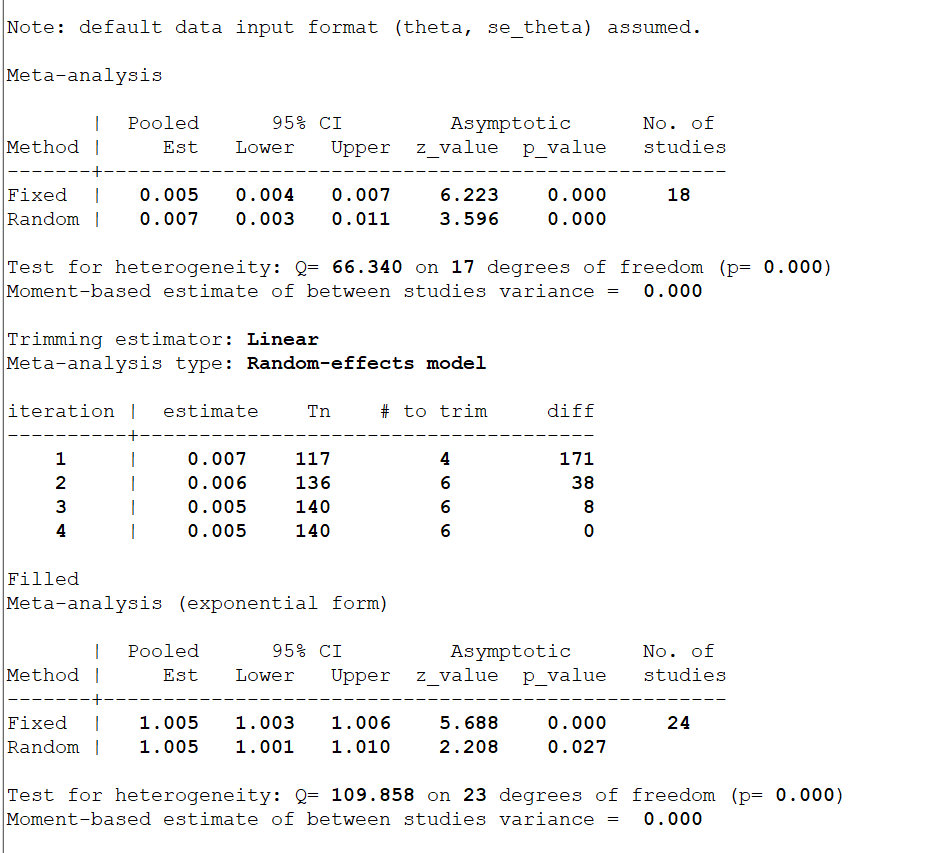


Figure S41 Results of Meta trim-and-fill for prolonged cardiopulmonary bypass.


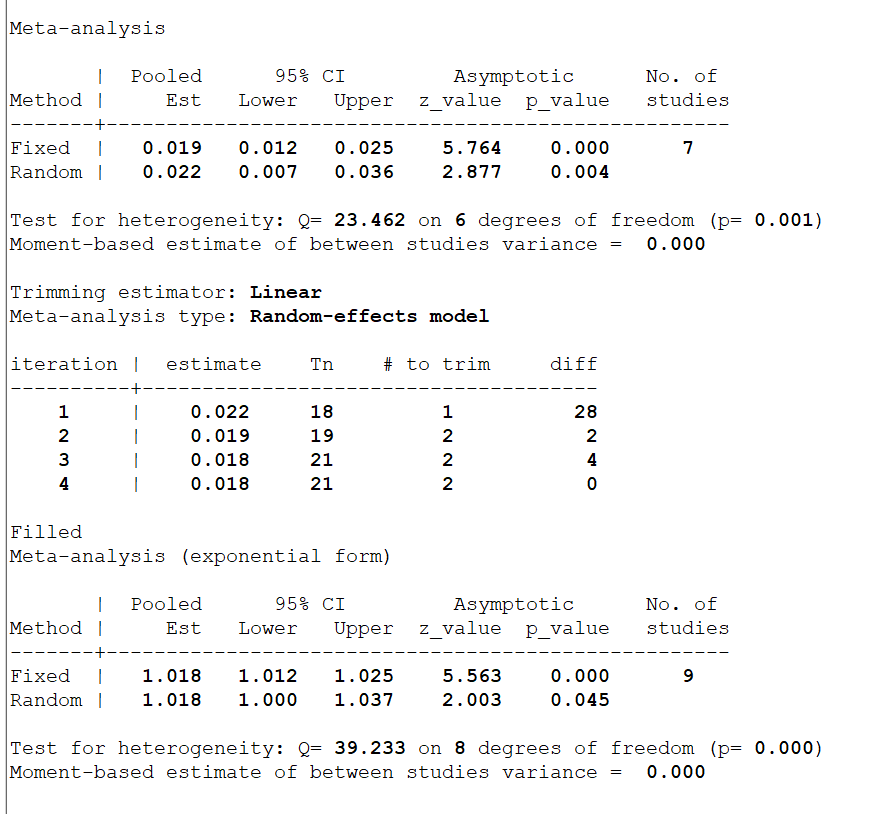
 Figure S42 Results of Meta trim-and-fill for preoperative serum creatinine.


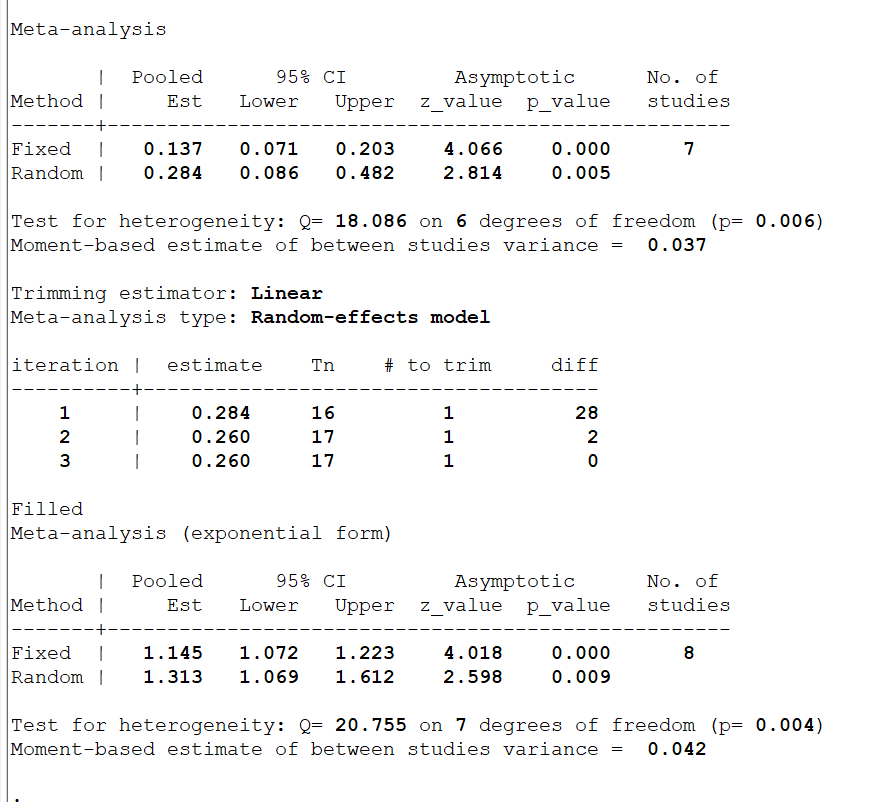
 Figure S43 Results of Meta trim-and-fill for prolonged duration of surgery.


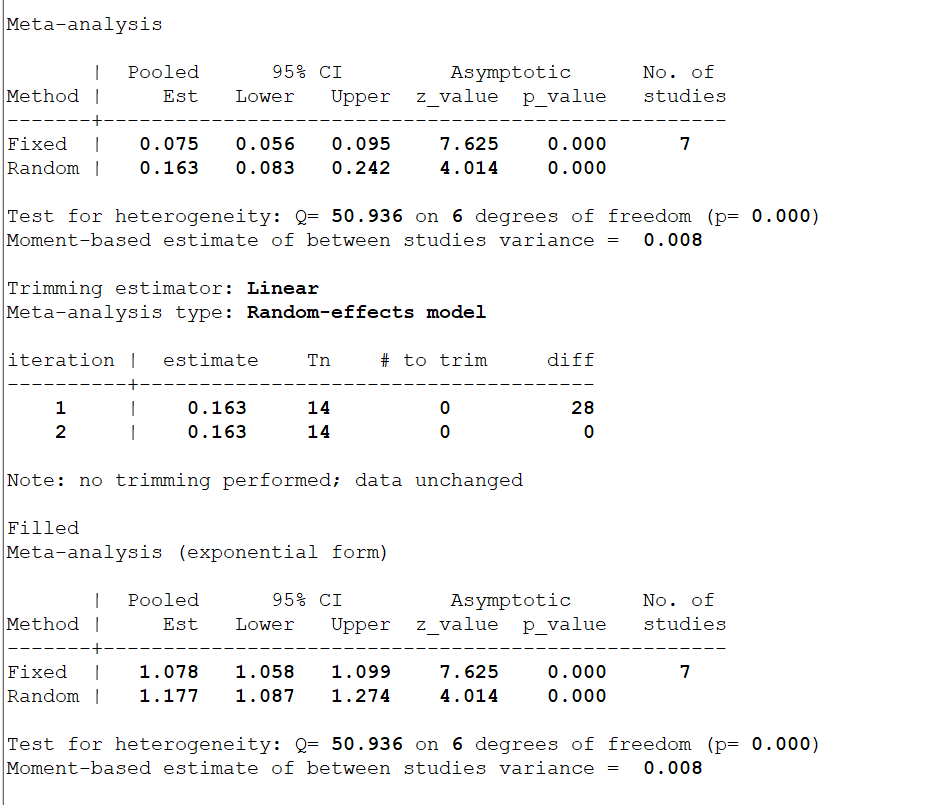
 Figure S44 Results of Meta trim-and-fill for the volume of red blood cell transfusion.


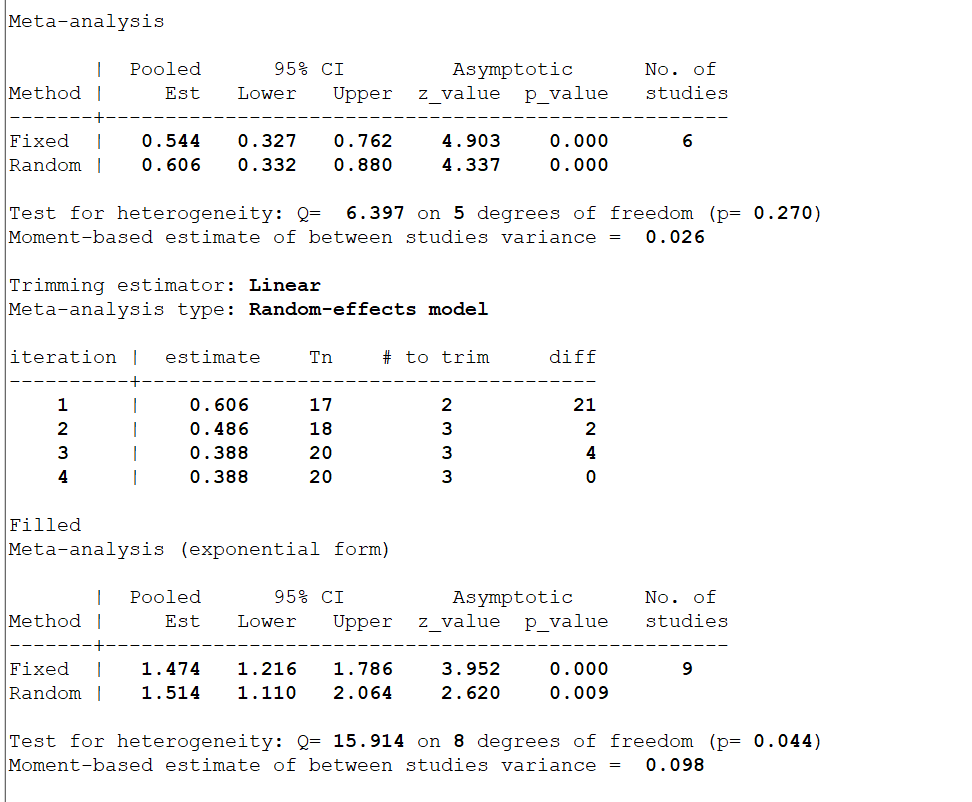


Figure S45 Results of Meta trim-and-fill for male.


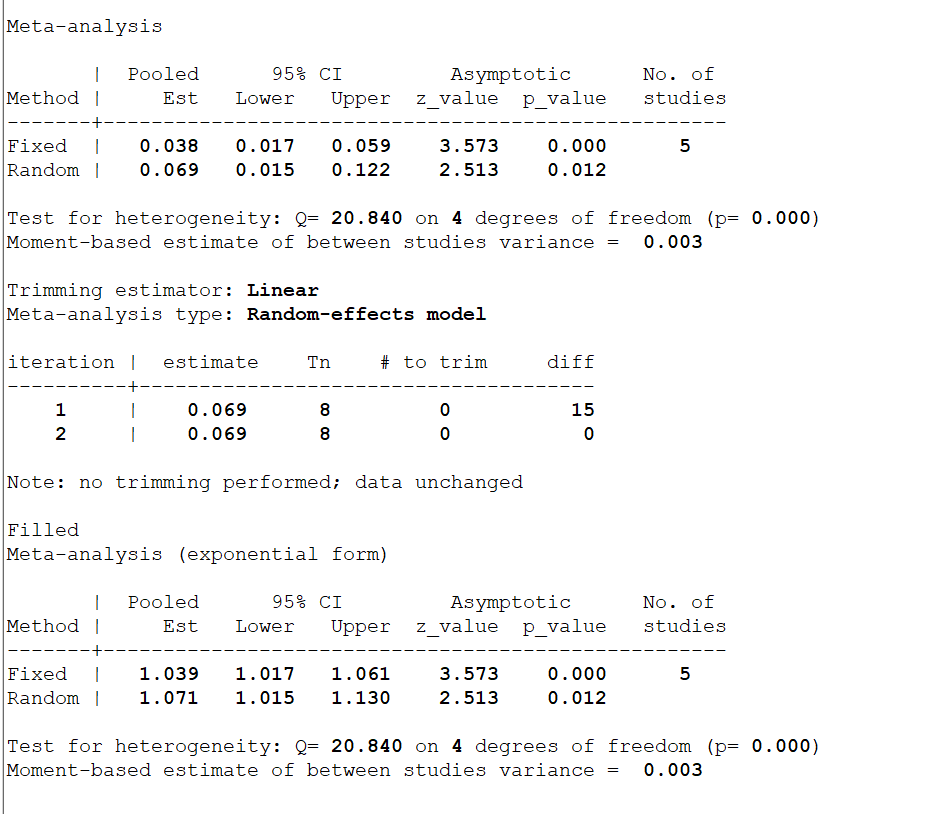
 Figure S46 Results of Meta trim-and-fill for duration of deep hypothermic circulatory arrest.
